# Influence of High-κ Dielectrics Integration on ALD-Based MoS_2_ Field-Effect Transistor Performance

**DOI:** 10.1021/acsanm.4c02214

**Published:** 2024-08-12

**Authors:** Reyhaneh Mahlouji, Yue Zhang, Marcel A. Verheijen, Saurabh Karwal, Jan P. Hofmann, Wilhelmus M. M. Kessels, Ageeth A. Bol

**Affiliations:** † Department of Applied Physics, Eindhoven University of Technology, P.O. Box 513, 5600 MB Eindhoven, The Netherlands; ‡ Laboratory of Inorganic Materials and Catalysis, Department of Chemical Engineering and Chemistry, Eindhoven University of Technology, P.O. Box 513, 5600 MB Eindhoven, The Netherlands; § Eurofins Materials Science, High Tech Campus 11, 5656 AE Eindhoven, The Netherlands; ∥ Surface Science Laboratory, Department of Materials and Earth Sciences, Technical University of Darmstadt, Otto-Berndt-Strasse 3, 64287 Darmstadt, Germany; ⊥ Netherlands Organization for Applied Scientific Research (TNO), 2628 CK Delft, The Netherlands; # Department of Chemistry, University of Michigan, 930 N. University Ave., Ann Arbor, Michigan 48109, United States

**Keywords:** high-κ dielectrics, atomic layer deposition, MoS_2_ field-effect transistors (FETs), charge transfer doping, current−voltage (*I*−*V)* characterization, XPS, STEM-EDX

## Abstract

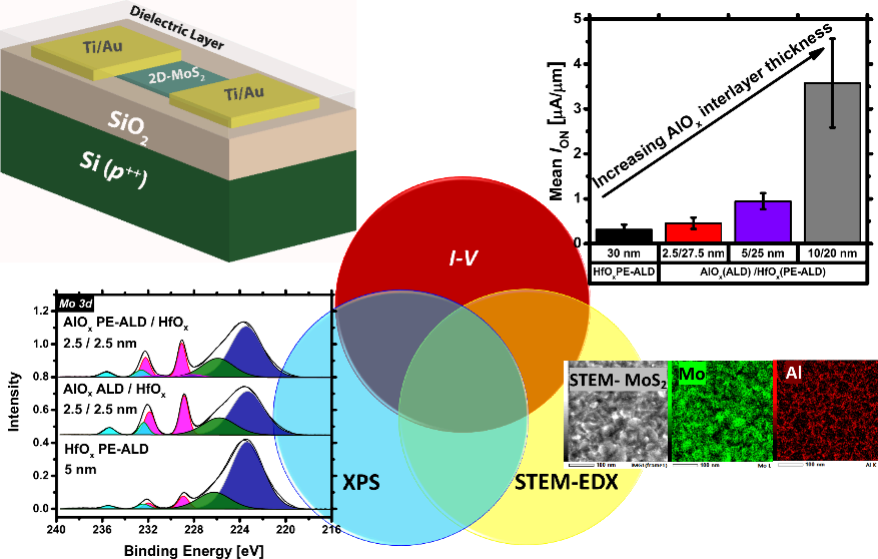

The integration of high-κ dielectrics on MoS_2_ field-effect transistors (FETs) is essential for the realization of MoS_2_ in ultrascaled nanoelectronic devices and circuits. Most studies covering this topic are based on exfoliated MoS_2_ flakes or chemical vapor deposition (CVD) grown MoS_2_ films, whereas other techniques, such as atomic layer deposition (ALD), are also gaining attention for the growth of MoS_2_ in recent years. In this work, we grow large-area MoS_2_ by means of plasma-enhanced (PE-)ALD and evaluate the influence of high-κ dielectrics on the properties of ALD-based MoS_2_ FETs through electrical characterization combined with surface-chemical and high-resolution scanning transmission electron microscopy (HR-STEM) analyses. We grow HfO_*x*_, AlO_*x*_, or both by means of PE-ALD or thermal ALD on our fabricated devices and show that, in addition to the dielectric constant, three other major parameters related to the processing of the dielectrics can simultaneously affect the MoS_2_ FET electrical characteristics and govern its doping. These parameters are the stoichiometry of the dielectric, its carbon impurity content, and the degree to which the MoS_2_ surface oxidizes upon the dielectric growth. When grown at 100 °C, our HfO_*x*_ films are oxygen-vacant whereas our AlO_*x*_ films are oxygen-rich. In addition, carbon impurities are incorporated into the dielectrics at low deposition temperatures, being one of the likely causes of the MoS_2_ FET overall *n*-type performance in all of the studied cases. Our investigations also reveal that PE-ALD of HfO_*x*_ or AlO_*x*_ oxidizes the MoS_2_ surface, whereas thermal ALD AlO_*x*_ leaves MoS_2_ almost intact. In this respect, if thermal ALD AlO_*x*_ of proper thickness is grown between MoS_2_ and HfO_*x*_, it can reduce the degree to which the MoS_2_ surface oxidizes by HfO_*x*_ and meanwhile improve the total dielectric constant, altogether leading to the most optimal electrical performance in ALD-based MoS_2_ FETs.

## 1 Introduction

Ever since layered two-dimensional (2D) transition metal dichalcogenides (TMDCs) were highlighted as potential material candidates for sub-10 nm technology nodes,^1−3^ there has been a growing interest in validating the feasibility of their implementation into such prospective platforms.^3−6^ Among the 2D TMDCs with semiconducting properties, MoS_2_ is the most extensively studied material, especially in the context of field-effect transistors (FETs). MoS_2_ FET device metrics are continually progressing, with the highest records being so far held for a current density of 700–1135 μA/μm,^7−9^ a subthreshold swing (*SS*) close to the thermionic limit (∼60 mV/dec),^10,11^ a field-effect mobility (μ_FE_) of 200 cm^2^/(V·s),^12^ and a low contact resistance (*R*_c_) range of 123–480 Ω·μm,^7,9,13^ although none of these figures of merits have been demonstrated all together in the same device. Besides these fascinating device properties, MoS_2_ FETs have demonstrated relatively low variability, good reproducibility, and decent reliability,^10,14,15^ as other vital conducts for their realization into more complex circuits.^10,16^ However, implementation of emerging materials on technology-relevant scales has its own challenges. As with semiconducting MoS_2_, there are yet several major hurdles that first need to be surmounted. Among them, the integration of nanometer-thin high dielectric constant (high-κ) materials with MoS_2_ FETs, as well as the large-area synthesis of high quality MoS_2_ films, is of crucial importance.

High-κ materials, such as HfO_2_, ZrO_2_, and Al_2_O_3_, are commonly used as gate dielectrics in FETs, with the aim of boosting the gate electrostatic control over the channel material.^17^ In conventional Si-based FETs, ultrathin layers of high-κ (<2 nm),^18^ which are necessary for downscaling the size of devices, can be successfully grown by means of atomic layer deposition (ALD). ALD is a cyclic thin film deposition technique that is well settled in industry and results in thickness-controlled, highly uniform, conformal and continuous films, free of pinholes.^19^ During the ALD process, layer growth is mainly governed by surface chemical reactions between the substrate of choice and the ALD reactants.

As far as the ALD growth of dielectrics on 2D MoS_2_ is considered, due to the all-surface and chemically inert basal planes of pristine MoS_2_, this process is mostly hampered by the lack of surface groups to which the ALD reactants could chemisorb.^20,21^ Therefore, nonuniform and defective dielectric films with incomplete surface coverages are often grown on pristine MoS_2_, especially for sub-10 nm high-κ thicknesses.^20−22^ Several attempts have been made to address this issue. UV/O_3_^23^ or O_2_ plasma^24^ surface functionalization and addition of a buffer/seed layer,^10,25,26^ prior to the high-κ deposition on MoS_2_, as well as employment of plasma-enhanced (PE)-ALD^27,28^ were overall beneficial in improving the growth quality of ultrathin films of these dielectrics on MoS_2_.

In addition to the gate dielectric role in FETs, high-κ materials can also act as protective or passivating layers for ultrathin nanomaterials and lead to further improvements in the electronic properties of the resulting devices.^12,29^ For example, capping MoS_2_ FETs with high-κ dielectrics, especially for back-gate devices, has been shown to highly influence the device metrics such as μ_FE_, the maximum drive current (*I*_ON_), and hysteresis.^12,14,30−32^ Theoretical studies have associated these improvements mainly with the efficient screening of MoS_2_ charged impurities (CIs) by the dipoles of high-κ materials.^33−35^ CIs are considered as the dominant Coulombic scattering centers in/on 2D materials, and they lead to carrier transport degradation.^33−35^ MoS_2_ CIs can have various sources, but they typically originate from intrinsic structural defects (e.g., Mo/S vacancies),^36^ environmental adsorbates (e.g., H_2_O),^14,37,38^ and organic residues formed during the device fabrication.^11,21^

Parallel to the suppression of CI scattering, high-κ materials have the capability of doping the underlying MoS_2_ as well. This is mainly attributed to the existence of bulk/interfacial fixed charges,^39,40^ which are formed when the dielectric film is sub(over)-stoichiometric and contains oxygen-vacant (oxygen-rich) states. The dielectric fixed charges can electrostatically induce carriers of opposite sign into the channel^31,41^ or dope the channel via a process known as charge transfer doping.^42^ These charges, furthermore, are shown to reduce the contact resistance (*R*_c_) and improve MoS_2_ conductivity by lowering Schottky barrier height (SBH) at the contact-to-MoS_2_ interface.^43^ Both experimental and theoretical studies have shown that the charge transfer doping process via oxygen-vacant (oxygen-rich) high-κ materials (e.g., HfO_*x*_, AlO_*x*_, and TiO_*x*_) can *n*-type dope (*p*-type dope) MoS_2_^7,26,44,45^ mainly because the defect energy bands in such films are distributed in the vicinity of MoS_2_ conduction band edge (*E*_c_) (valence band edge (*E*_v_)).^7,44^ Therefore, tuning the dielectric film stoichiometry (as recently shown^43,46^) can be a way forward for controllably doping the MoS_2_ films and improving their electronic properties. In addition to the oxygen-vacant (oxygen-rich) states, the carbon content in high-κ dielectrics is also another doping contributor,^47^ wherewith the carrier density and other MoS_2_ FET properties can be adjusted. All of these investigations highlight the determining role of the dielectrics in tailoring the electrical characteristics of MoS_2_ FETs.

As mentioned earlier, synthesis of high quality MoS_2_ films over large areas is the other challenge for the realization of MoS_2_ FETs in ultrascaled circuits. Although chemical vapor deposition (CVD) is one of the primary techniques to fulfill this demand,^48^ in pursuit of a low thermal budget (required for industrial processes) as well as a precise thickness control, (PE-)ALD has gained attention in recent years for the growth of MoS_2_ and other 2D TMDCs.^49−51^ However, films obtained by this technique are polycrystalline, with point defects and grain boundaries (GBs) inherent to their synthesis route.^52^

As far as the growth of high-κ dielectrics on such synthetic and polycrystalline MoS_2_ films is concerned, it has been shown that the dielectric layer mostly nucleates along the MoS_2_ GBs and defect sites. This leads to the growth of dielectric nanoribbons^53^ during the initial stages of the dielectric deposition and subsequently results in an incomplete MoS_2_ surface coverage for sub-10 nm thicknesses.^53,54^ Therefore, it can be inferred that the growth of ultrathin, high-quality, and highly uniform dielectrics on polycrystalline MoS_2_ has its own challenges, which can be different from the pristine MoS_2_ films obtained by exfoliation. Up until now, the integration of high-κ materials has been mainly studied on exfoliated MoS_2_ flakes^12,21,27^ or large-grain CVD grown MoS_2_ films,^7,28,32^ with no studies directly evaluating the growth of dielectrics on ALD-based MoS_2_ films.

In this study, we employ an ALD-based approach for the large-area synthesis of MoS_2_.^49,55^ Because of the principal differences between the morphology of ALD-based MoS_2_ films and those obtained by other techniques (e.g., smaller grain size, more GBs, and possibly deviating stoichiometries), the influence of the commonly used dielectrics on the electrical properties of ALD-based MoS_2_ films needs to be investigated independently. In this contribution, we grow AlO_*x*_, HfO_*x*_, or combinations thereof on ALD-based MoS_2_ FETs. Through current–voltage (*I–V*) characterization, we evaluate the electrical performance of the fabricated devices. We link our electrical findings to the chemistry involved at the high-κ/MoS_2_ interface by means of X-ray photoelectron spectroscopy (XPS) analysis. We also assess the dielectric film coverage on MoS_2_ in the early cycles of high-κ deposition using top view scanning transmission electron microscopy (STEM) imaging.

## 2 Experimental Section

### 2.1 MoS_2_ Film Synthesis

For this work, 7–8 monolayers of MoS_2_ were synthesized by a two-step approach, where large-area and thickness-controlled MoO_*x*_ films were first grown at 50 °C using PE-ALD^56^ in an Oxford Instruments Plasma Technology ALD reactor (FlexAL). The as-deposited MoO_*x*_ films, with a thickness of ∼4.9 nm, were then annealed in a tube furnace, where a H_2_S/Ar gas mixture of 10%/90% was introduced at 900 °C for 45 min, to eventually obtain MoS_2_ multilayers. Further details of the synthesis method and film specifications are published in a previous work.^55^ In all cases, the film growth took place on highly doped (*p*^++^) Si substrates, thermally coated with ∼285 nm SiO_2_, serving as a global back-gate for the fabricated MoS_2_ FETs.

### 2.2 Back-Gate Device Fabrication

The back-gated MoS_2_ FETs were fabricated using a standard two-step electron beam lithography (EBL) technique. Details of the device fabrication are reported in a previous study.^57^ In this work, a combination of 5/95 nm Ti/Au was electron beam evaporated as the contacts to MoS_2_. Then, to define the channel areas, MoS_2_ was dry etched using a SF_6_/O_2_ plasma gas mixture. The fabricated devices were finally capped with a 30 nm thick high-κ dielectric layer. A top-view optical microscopy image of the final MoS_2_ FETs and cross-sectional schematics are provided in Figures S1a and S1b, respectively.

### 2.3 MoS_2_ FET Capping with PE-ALD Grown HfO_*x*_

Investigations regarding the influence of dielectrics on the back-gate MoS_2_ FETs were initiated with the growth of 30 nm HfO_*x*_ on the fabricated devices using the PE-ALD method at three different table temperatures of 100, 200, and 300 °C. HfCp(NMe_2_)_3_ (HyALD) and O_2_ plasma were the precursor and the oxygen agent used during this process, respectively.^58^ As HyALD precursor does not react with H_2_O, films of HfO_*x*_ could only be grown using the PE-ALD method, and thermal ALD of HfO_*x*_ could not be examined in this study. Further details and specifications of the growth process are provided in Section S.2.1 of the Supporting Information.

### 2.4 MoS_2_ FET Capping with ALD and PE-ALD Grown AlO_*x*_

Next, 30 nm thick AlO_*x*_ dielectrics were grown on the fabricated MoS_2_ FETs using both ALD and PE-ALD processes at 100 °C.^59^ We note that throughout this study ALD refers to thermal ALD. For the ALD of AlO_*x*_, trimethylaluminum (TMA, Al(CH_3_)_3_) vapor and H_2_O were employed as the precursor and the oxygen agent, respectively, and for the PE-ALD of AlO_*x*_, TMA and O_2_ plasma were utilized. See the Supporting Information for further details of both methods and their processing conditions (Sections S.2.2 and S.2.3).

### 2.5 MoS_2_ FET Capping with ALD/PE-ALD Grown Bilayers of AlO_*x*_/HfO_*x*_

Bilayer stacks of AlO_*x*_/HfO_*x*_ were also evaluated as capping layers for the MoS_2_ FETs. The processing conditions were similar as to when the AlO_*x*_ and HfO_*x*_ films were grown individually on the fabricated devices at 100 °C. The thickness of the entire dielectric stack was maintained at 30 nm, while examining 2.5/27.5 nm, 5/25 nm, and 10/20 nm of AlO_*x*_/HfO_*x*_ stack combinations. The AlO_*x*_ interlayer was grown by both ALD and PE-ALD methods, except in the case of the 10/20 nm combination where only ALD was used for the growth AlO_*x*_.

### 2.6 Dielectric Thickness Measurements

The data for the high-κ film thicknesses were collected using an in situ spectroscopic ellipsometry (SE) instrument (J.A. Woollam Co., Inc. M-2000F, 1.24–5 eV range). The SE measurements were conducted on a control sample of 450 nm SiO_2_/Si. We assume that the growth of dielectrics on MoS_2_ is approximately the same as that on SiO_2_/Si substrates. The film thicknesses were obtained using a Cauchy-based fitting model in Complete EASE software.

### 2.7 Electrical Characterization

The room-temperature electrical measurements were performed on 500 nm long and 1 μm wide MoS_2_ channels using a Keithley 4200-SCS parameter analyzer, which was connected to a cryogenic probe station (Janis ST-500) operating at a base pressure of ∼1.9 × 10^–4^ mbar.

### 2.8 Surface Characterization

The chemistry involved at the high-κ dielectric/MoS_2_ interface was investigated using ex-situ XPS. For this purpose, the dielectric films were grown on blanket films of MoS_2_ with equal deposition conditions as to when they were grown on the fabricated MoS_2_ FETs, so that consistency could be maintained. The setup used for the XPS analyses was a Thermo Scientific K-alpha KA1066 spectrometer (Thermo Fisher Scientific, Waltham, MA) with a monochromatic Al Kα X-ray radiation source (*hv* = 1486.6 eV). The measurements were carried out with an X-ray beam spot size of 400 μm, at a takeoff angle of 60°, and a pass energy of 50 eV combined with an electron flood gun to efficiently neutralize the existing charges on the samples as well as to correct for the nonuniform/differential charging. The acquired spectra were later chemically quantified and deconvoluted with Avantage software. All the binding energies (BEs) were calibrated with respect to the C 1s adventitious carbon peak, set to 284.8 eV.

### 2.9 Structural Characterization

The high-κ coverage on MoS_2_ was also studied using a top-view STEM imaging with a JEOL atomic resolution microscope (ARM) 200-F, operated at 200 kV and equipped with a 100 mm^2^ Centurio silicon drift detector (SDD) as well as an energy dispersive X-ray (EDX) detector. The analyses took place during the early cycles of (PE-)ALD, and electron transparent SiN_*x*_ TEM windows, coated with ∼5 nm ALD SiO_2_, were utilized as the imaging substrates. The MoS_2_ films were grown on these substrates under equal conditions as when they were being synthesized for devices (explained earlier), followed by relevant dielectric depositions.

## 3 Results and Discussion

### 3.1 HfO_*x*_ Capping Layer

As already mentioned, high-κ materials can efficiently screen the MoS_2_ CIs^33−35^ and electrostatically dope this 2D layer.^7,26,44,45^ HfO_*x*_ is one of the most studied dielectrics in the context of FETs. Throughout this section, we evaluate the influence of HfO_*x*_ on the electrical performance of our ALD-based MoS_2_ FETs.

#### 3.1.1 Electrical Characterization of HfO_*x*_-Capped MoS_2_ FETs

**Figure 1:**
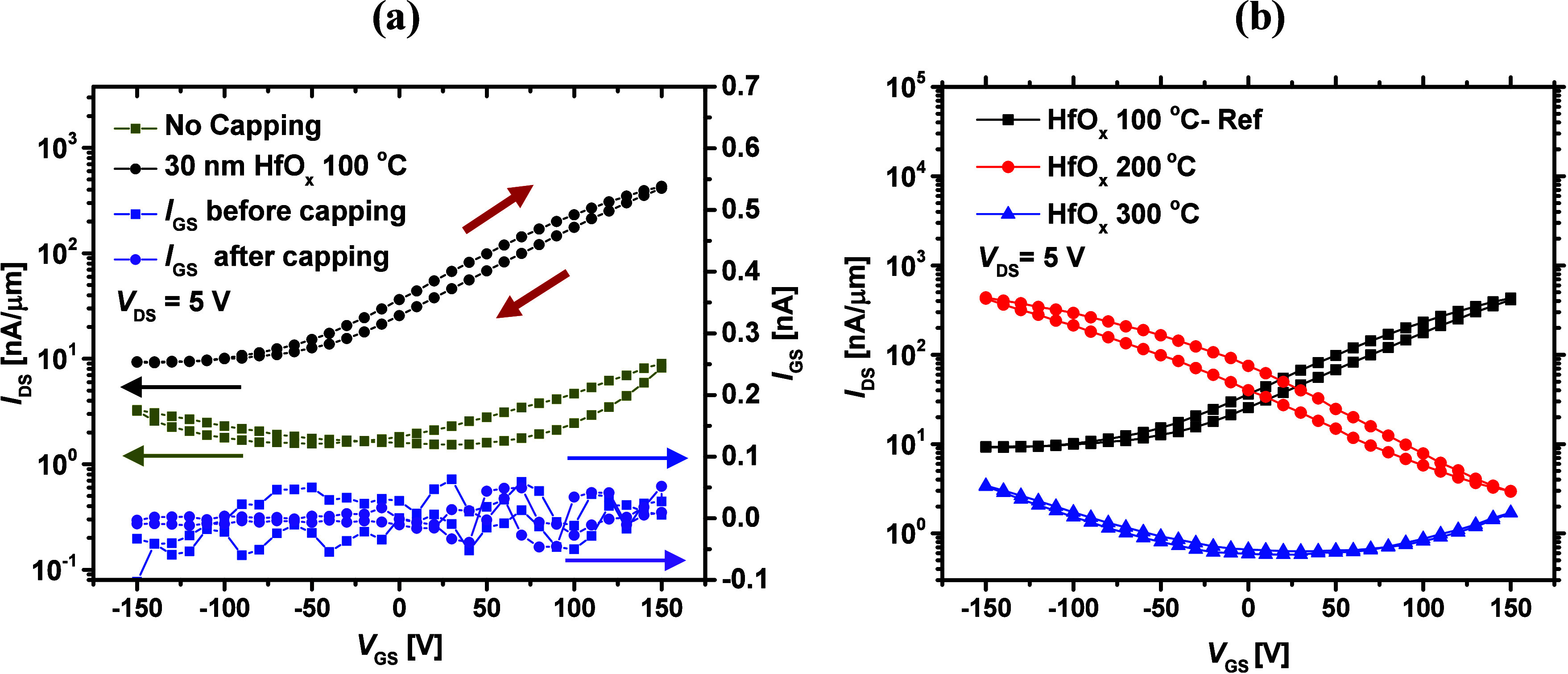
(a) Left axis shows the transfer curves of a device with and without HfO_*x*_ grown at 100 °C (on a semilog scale). The red arrows determine the direction of forward and backward current sweeps. The right axis shows *I*_GS_ in linear scale, (b) transfer curves of the MoS_2_ FETs capped with HfO_*x*_ films grown at deposition temperatures of 100, 200, and 300 °C.

Initially, 30 nm HfO_*x*_ was grown on the back-gate MoS_2_ FETs using PE-ALD^58^ at 100 °C. Figure 1a (left axis) shows the normalized double sweep transfer characteristics (*I*_DS_–*V*_GS_) of a device with and without HfO_*x*_. As can be seen, upon capping of the MoS_2_ FETs with HfO_*x*_, the device metrics improve significantly. The maximum drain current (*I*_ON_) increases at least 50 times and reaches 0.5 μA/μm, the current rise in the *p*-branch is suppressed, and the hysteresis reduces. However, the minimum drain current (*I*_OFF_) slightly increases, and the threshold voltage (*V*_T_) shifts to more negative values.

These observations imply that the MoS_2_ channel becomes *n*-type doped upon the PE-ALD of HfO_*x*_ at 100 °C. In addition, the hysteresis reduction could be an indication of environmental adsorbates (e.g., water molecules) and defective states in the MoS_2_. By capping MoS_2_ with HfO_*x*_, environmental adsorbates are prevented, and the density of defective states on MoS_2_ surface could be reduced.^14,30,32^ It is also important to evaluate the gate leakage current (*I*_GS_) of the fabricated FETs. As shown in the right axis of Figure 1a, before and after the device capping, *I*_GS_ is maintained well below 0.1 nA, inferring that no additional conductive path other than the MoS_2_ channel is present in our fabricated devices. All throughout this work, we consider the MoS_2_ FETs capped with PE-ALD grown HfO_*x*_ at 100 °C as our reference device. Hence, the results obtained from other fabricated devices are compared with this case.

Next, the influence of the HfO_*x*_ growth temperature on the electrical performance of the devices was investigated. For this purpose, 30 nm HfO_*x*_ films were grown on the as-fabricated MoS_2_ FETs at 200 and 300 °C, while keeping all other PE-ALD conditions equal to the reference. As provided in Figure 1b, increasing the HfO_*x*_ deposition temperature changes the MoS_2_ FET transfer characteristics. Unlike the reference, complete *p*-type device behavior is observed upon HfO_*x*_ growth at 200 °C, with *I*_ON_ reaching 0.5 μA/μm. When HfO_*x*_ is deposited at 300 °C, a highly degraded performance is noticed, with a minor rise in the *p*-branch.

Based on a former report by Sharma et al.,^58^ our PE-ALD grown HfO_*x*_ films are substoichiometric and contain oxygen vacancies if grown below 400 °C. The oxygen vacancy states in a substoichiometric dielectric (e.g., HfO_*x*_, AlO_*x*_, and TiO_*x*_) are known to be energetically distributed in close proximity to the MoS_2_*E*_c_,^7,26,44,45^ and introducing only 0.9% of oxygen vacancies into these dielectrics has been shown to significantly *n*-dope MoS_2_.^32^ Therefore, MoS_2_ FETs can become readily *n*-type doped, and *R*_c_ can be reduced^43^ by the transfer of charges from the dielectric oxygen vacancy states to the MoS_2_ + *E*_c_.^60^ Sharma et al. have also demonstrated that the HfO_*x*_ film stoichiometry depends on the deposition temperature, and increasing the temperature from 100 to 300 °C results in the HfO_*x*_ films that are closer to full stoichiometry.^58^

These earlier findings can clearly explain the observed *n*-type device characteristics in our reference case, as the HfO_*x*_ capping layer grown at 100 °C contains the most oxygen vacancies among the three studied cases. However, the electrical behavior observed for the devices capped with HfO_*x*_ films grown at 200 and 300 °C cannot be attributed to the presence of oxygen vacancies. With increasing the growth temperature, the HfO_*x*_ films get closer to full stoichiometry^58^ and, hence, lead to less *n*-type doping. This should manifest itself in a relative *I*_OFF_ reduction and a positive shift of *V*_T_. However, a complete *p*-type device behavior and a decaying performance are noticed for the MoS_2_ FETs, with sequentially increasing the HfO_*x*_ growth temperature. To explain these electrical observations, the chemistry involved at the HfO_*x*_/MoS_2_ interface needs to be investigated in the next step.

#### 3.1.2 XPS Analysis of HfO_*x*_-Capped MoS_2_

For this purpose, ex-situ XPS measurements were conducted before and after the growth of ∼2.5 nm HfO_*x*_ on bare (as-synthesized) MoS_2_ films at 100, 200, and 300 °C. Because of the limited depth resolution of XPS tools (∼7 nm), only 2.5 nm of HfO_*x*_ was deposited in this phase, so that the HfO_*x*_/MoS_2_ interface could be well evaluated. Depth profiling of the stack was also not an option due to the preferential sputtering of S.

**Figure 2:**
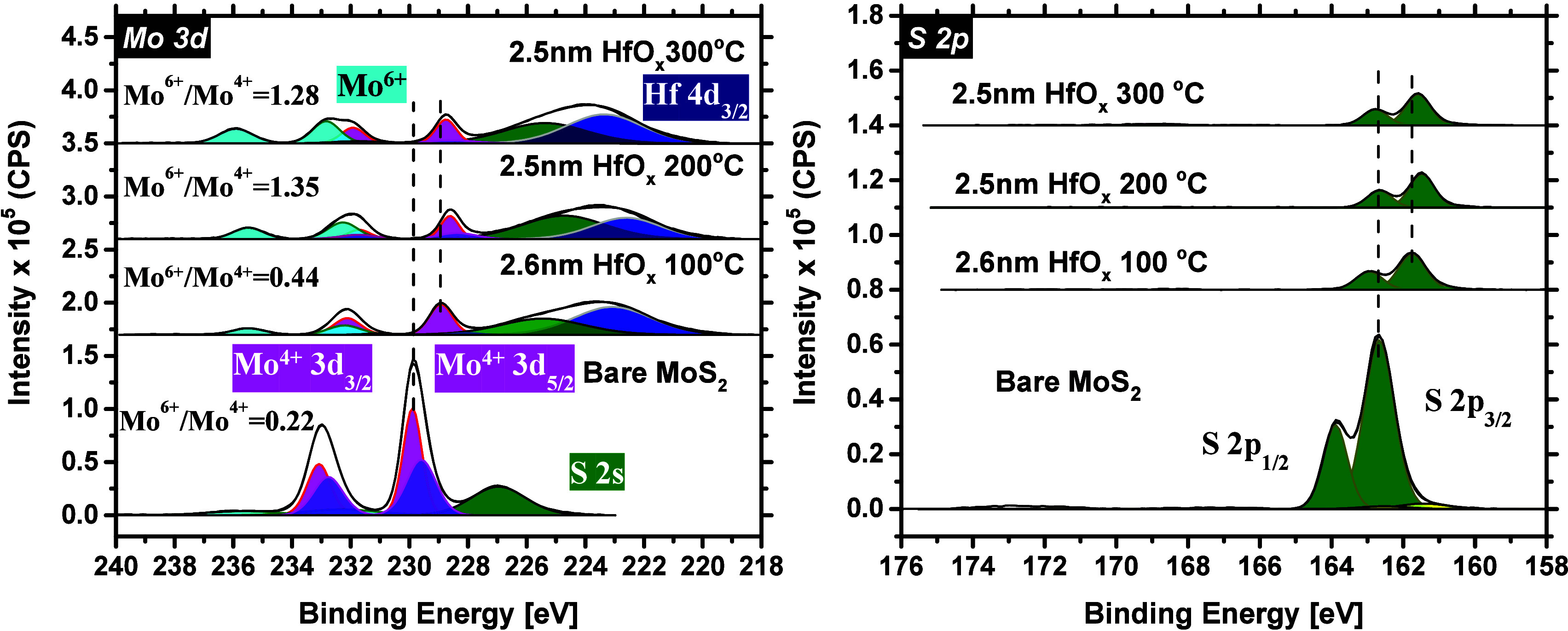
Mo 3d and S 2p core level spectra before and after the deposition of 2.5 nm HfO_*x*_ at 100, 200, and 300 °C. Panels in each plot are offset vertically with respect to the bare MoS_2_.

Figure 2 shows the acquired elemental data for the core level spectra of the Mo 3d and S 2p, fitted accordingly, and charge corrected against the C 1s adventitious peak (284.8 eV) (for the C 1 s spectrum, see Figure S2). The bottom panel in each plot corresponds to the bare MoS_2_ film with no dielectric capping, and the rest of the panels are offset vertically. Considering the Mo 3d core level spectrum of the bare MoS_2_ case, two major peaks (as representatives of the Mo^4+^ 3d_5/2_ and Mo^4+^ 3d_3/2_ doublet peaks) are detected at 229.8 and 233.0 eV, respectively.

To improve the fitting, another doublet was also required, with peaks of slightly lower BEs than the Mo^4+^ doublet, proposing that our synthetic MoS_2_ film is slightly substoichiometric and sulfur-deficient.^61^ At the left side of this spectrum (higher BEs), the Mo^6+^ oxidation state (MoO_*x*_, *x* ∼ 3) is also detected, which is mainly due to the exposure of the MoS_2_ surface to the ambient air. Finally, as a result of the S 2s core level overlapping into the Mo 3d spectrum, an extra peak is detected at a lower BE of 226.9 eV. Considering the S 2p spectrum, the S 2p_3/2_ and S 2p_1/2_ doublets are identified at 162.6 and 163.8 eV, respectively.

Upon the growth of ∼2.5 nm of HfO_*x*_ on MoS_2_ at 100 °C, the intensity of the Mo 3d and S 2p major peaks drops considerably. In addition, a new peak appears at 223.0 eV, which is attributed to the Hf 4d_3/2_ chemical state. The integrated area under the Mo^6+^ doublet peaks slightly increases as well, and the Mo^4+^ doublet peak BEs shift −1.00 eV (relative to the Mo^4+^ BEs of bare MoS_2_). A similar magnitude of the BE shift is also detected for the S 2p core levels. These observations altogether imply that upon the growth of HfO_*x*_, the concentration of MoO_*x*_ (*x* ∼ 3) species increases on the MoS_2_ surface and the MoS_2_ surface oxidizes. This is expected since O_2_ plasma is used as the oxygen agent during the growth of HfO_*x*_, and the MoS_2_ surface is exposed to it from the beginning of the PE-ALD process.

Elevating the growth temperature of HfO_*x*_ to 200 and 300 °C, the Mo^6+^ doublet peaks shift to higher BEs, and the integrated area under these peaks further increases. The Mo^6+^/Mo^4+^ ratio changes from 0.44 (at 100 °C) to 1.35 (at 200 °C) and to 1.28 (at 300 °C). Details of the Mo^6+^ and Mo^4+^ doublet peak areas for all the studied cases are provided in Table S1. In addition, with increasing the growth temperature to 200 and 300 °C, both the Mo 3d and S 2p major peaks shift −1.3 and −1.1 eV, respectively, as compared with the bare MoS_2_ case. These findings are jointly pointing in the direction that when HfO_*x*_ is deposited at temperatures higher than 100 °C, the concentration of MoO_*x*_ (*x* ∼ 3) increases considerably, and that the MoS_2_ surface further oxidizes, mainly because of higher surface reactivity at elevated temperatures.

It is also important to verify that (part of) the MoS_2_ films are still structurally intact upon the growth of HfO_*x*_. For this purpose, a Raman analysis was performed. The data provided in Figure S3 reveal that the characteristic MoS_2_ Raman modes (A_1g_ and E^1^_2g_ peaks) are still present after the growth of HfO_*x*_, irrespective of the HfO_*x*_ deposition temperature.

#### 3.1.3 Linking XPS Results to *I–V* Characterization

To correlate the XPS chemical findings with the *I–V* measurements, it is essential to first point out that the MoO_*x*_ compound detected on the MoS_2_ surface is generally known as a hole dopant for MoS_2_, given that *x* ≤ 3.^46,62^ The MoO_*x*_ bandgap (*E*_g_) reaches up to ∼6.5 eV, and the oxygen vacancy states in MoO_*x*_ energetically reside close to the MoS_2_*E*_v_.^62^

Based on the XPS data, when the HfO_*x*_ is grown at 200 °C on MoS_2_, the concentration of MoO_*x*_ (*x* ∼ 3) increases significantly compared to when HfO_*x*_ is grown at 100 °C. As a result, more holes can be transferred/induced into the underlying MoS_2_. This could explain the *p*-type conductivity observed for the MoS_2_ FETs capped with the HfO_*x*_ film grown at 200 °C.

It is also expected that by raising the growth temperature of HfO_*x*_ further to 300 °C, the *p*-type doping effect in the MoS_2_ FETs could become more pronounced due to the additional increase in the MoO_*x*_ concentration. Although from XPS and Raman spectroscopy it can be concluded that there is still intact MoS_2_ under HfO_*x*_, the damage to the MoS_2_ surface (created by the O_2_ plasma usage during the HfO_2_ PE-ALD process) can be a likely cause for the observed device deterioration. Furthermore, one should note that the PE-ALD process of HfO_*x*_ takes place in the end of the device fabrication, when the contacts are delineated and the MoS_2_ channel is already defined. The growth of HfO_*x*_ at an elevated temperature of 300 °C can impose an additional annealing effect to the Ti/Au contacts. This is undesired, as previous studies have shown that annealing the Ti/Au contacts degrades the MoS_2_ FET electrical performance and increases *R*_c_.^12,37,57^ Therefore, it is speculated that the observed drop in the device metrics could also be attributed to the Ti/Au interface quality that deteriorates upon the growth of HfO_*x*_ at 300 °C. In addition to the MoS_2_ surface damage and contact annealing effect, the HfO_*x*_ films grown at elevated temperatures (>200 °C) usually show higher degree of crystallinity and subsequently higher surface roughness.^27,58^ The increase in the HfO_*x*_ surface roughness can increase the carrier scattering in MoS_2_.^27^ This can also contribute to the observed degradation in the MoS_2_ device performance. The effects mentioned above can altogether explain why the growth of HfO_*x*_ at 300 °C does not lead to further *p*-type doping of the MoS_2_ FETs.

In summary, the dielectric growth temperature strongly influences the ALD-based MoS_2_ FET characteristics. PE-ALD of HfO_*x*_ at 100 °C is oxygen-vacant and leads to an optimal *n*-type performance in the fabricated devices. When HfO_*x*_ is deposited at 200 °C, the MoS_2_ surface oxidizes and results in the observed *p*-type conductivity. Then, by growing HfO_*x*_ films at 300 °C, the MoS_2_ surface damage, the Ti/Au contact annealing effect, and the HfO_*x*_ surface roughness increase can simultaneously contribute to the fully deteriorated device performance.

### 3.2 AlO_*x*_ Capping Layer

In the next step, 30 nm AlO_*x*_ capping layers were grown on the fabricated MoS_2_ FETs, using both ALD and PE-ALD processes. A deposition temperature of 100 °C was chosen to allow for direct comparison with the reference (HfO_*x*_ case) and to prevent device degradation when the dielectric layer is grown at higher temperatures. PE-ALD can have several advantages over ALD, e.g., improved film properties and the possibility to grow the dielectrics at lower deposition temperatures. However, as seen in the previous section and observed in other studies, PE-ALD can induce unwanted damages to the underlying layer.^63^ As far as MoS_2_ is concerned, by carefully choosing the PE-ALD processing conditions, a uniform coverage of ultrathin dielectric films (AlO_*x*_ or HfO_*x*_) can be achieved on exfoliated MoS_2_ and without severely damaging this 2D layer.^27^ However, the growth of AlO_*x*_ with both ALD and PE-ALD as well as its nucleation behavior on the ALD-based polycrystalline MoS_2_ has not yet been thoroughly reported in the literature. Therefore, both methods are investigated for the growth of AlO_*x*_ on our fabricated devices.

#### 3.2.1 *I–V* Characterization of AlO_*x*_-Capped MoS_2_ FETs

**Figure 3:**
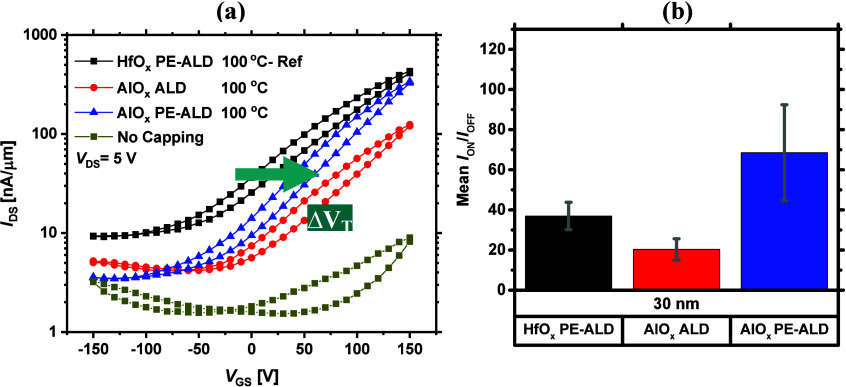
(a) Semilog double sweep transfer curves of the MoS_2_ FETs capped with AlO_*x*_ films, grown by ALD, PE-ALD at 100 °C, as well as the uncapped case. The arrow determines the relative change in *V*_T_ upon the growth of AlO_*x*_ on the fabricated devices. (b) Average *I*_ON_/*I*_OFF_ ratio after the growth of these dielectrics.

Figure 3a shows the transfer characteristics of AlO_*x*_-capped MoS_2_ FETs and compares the data with the reference device (capped with HfO_*x*_ grown by PE-ALD at 100 °C). As can be seen, irrespective of the deposition method, AlO_*x*_ capping leads to an overall *n*-type performance in MoS_2_ FETs. However, compared with the reference, in both AlO_*x*_-capped devices, *V*_T_ shifts positively and *I*_OFF_ reduces, implying a reduced *n*-type doping concentration in MoS_2_. As far as the AlO_*x*_ growth methods are concerned, a higher *I*_ON_ is achieved when AlO_*x*_ is grown by PE-ALD on MoS_2_ FETs. This could be attributed to the generally higher dielectric constant of PE-ALD AlO_*x*_ films compared to thermal ALD AlO_*x*_ films.^64^ A higher dielectric constant is known to screen the MoS_2_ CIs and reduce the free carrier scattering more effectively,^34^ thereby improving the MoS_2_ FET ON-current density. The average data shown in Figure S4a and obtained after measuring 3–4 devices at different locations on the individual samples also confirm an overall higher *I*_ON_ for the PE-ALD AlO_*x*_ case than the ALD counterpart. Among these three studied cases, PE-ALD AlO_*x*_-capped devices exhibit the most optimal performance due to their lower *I*_OFF_ and higher *I*_ON_/*I*_OFF_ ratio. The average data for *I*_ON_/*I*_OFF_ are provided in Figure 3b, and other device metrics (mean values for the maximum μ_FE_ and *I*_OFF_) are shown in Figure S4b,c, respectively.

One may assign the as-observed MoS_2_ FET overall *n*-type behavior to the AlO_*x*_ oxygen vacancy states.^7,44^ However, earlier studies^59,64^ have shown that our AlO_*x*_ films grown below 200 °C (with both ALD and PE-ALD methods) are oxygen-rich ([O]/[Al] > 1.5). The high oxygen content at low deposition temperatures is mainly associated with the incorporation of a considerable concentration of hydroxyl (−OH) groups into the films. The AlO_*x*_ used for capping our MoS_2_ FETs is grown at 100 °C, and our films are oxygen-rich. Therefore, the observed *n*-type device performance cannot be the result of oxygen vacancies.

A recent study has addressed this controversy by highlighting that the AlO_*x*_ carbon content can play a significant role in *n*-type doping the MoS_2_ FETs.^47^ Generally, ALD of dielectrics by metal–organic precursors at low deposition temperatures leads to carbon impurities into the films due to inefficient ligand abstraction.^65^ The carbon-related states are energetically distributed inside the AlO_*x*_*E*_g_ and close to the oxide midgap,^47^ such that they can donate electrons to the MoS_2_*E*_c_ via charge transfer doping process. Since we grow the AlO_*x*_ films at 100 °C, carbon impurities are anticipated. Therefore, one potential interpretation for the overall *n*-type behavior of our AlO_*x*_-capped MoS_2_ FETs can be the incorporation of carbon impurities into the AlO_*x*_ films. As far as the AlO_*x*_ oxygen-rich states are concerned, their role is to reduce the MoS_2_*n*-type doping density, since they transfer holes to the MoS_2_*E*_v_.^7,44^ This has been manifested in the positive shift of *V*_T_ and the reduction of *I*_OFF_ (relative to the reference case).

#### 3.2.2 XPS Analysis of AlO_*x*_-Capped MoS_2_ Films

**Figure 4:**
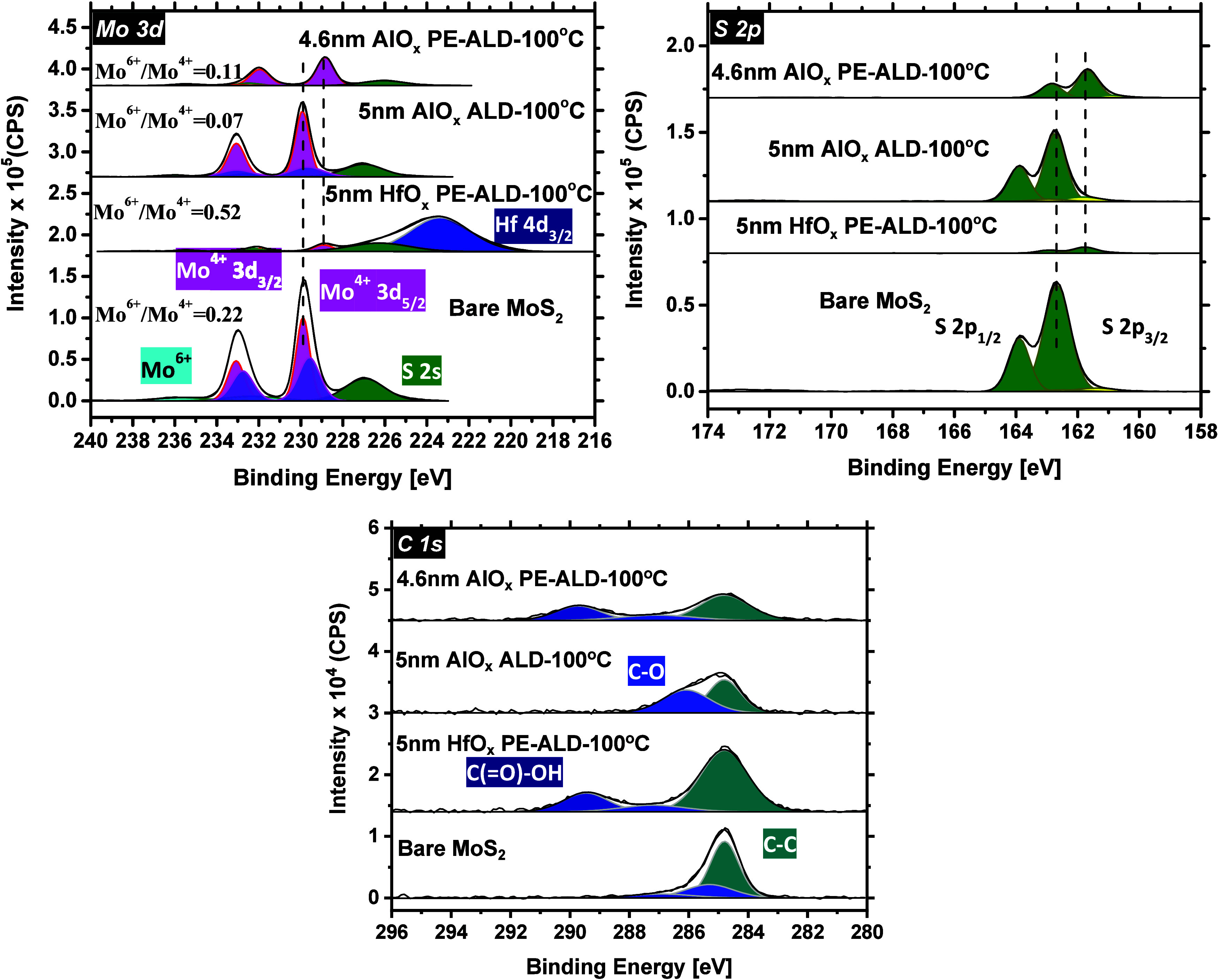
Mo 3d, S 2p, and C 1S core level spectra before and after the growth of ∼5 nm ALD and PE-ALD AlO_*x*_ as well as PE-ALD HfO_*x*_, all at the deposition temperature of 100 °C.

To investigate the carbon content and other chemical states at the dielectric/MoS_2_ interface, XPS analysis was performed before and after the growth of ∼5 nm of AlO_*x*_ on bare MoS_2_ films. Since the HfO_*x*_ capping layer is a reference for all of our analyses in this study, a comparable thickness of HfO_*x*_ on MoS_2_ is also chemically analyzed with XPS. Figure 4 shows the acquired elemental data for the Mo 3d, S 2p, and C 1s core level spectra, with the bottom panel in each plot as a representative of the bare MoS_2_.

Compared with the bare MoS_2_ case, after the growth of ALD AlO_*x*_ on MoS_2_, the peaks associated with the Mo^4+^ doublet remain unchanged in the Mo 3d spectrum, and no significant oxidized species are detected (the Mo^6+^/Mo^4+^ is only 0.07 (see Table S2)). In addition, neither the Mo 3d nor the S 2p major peak intensities drop dramatically. These observations imply that the ALD of AlO_*x*_ does not oxidize the MoS_2_ surface and that only a very thin layer of AlO_*x*_ is deposited on MoS_2_ (as also verified by the Al 2p spectrum). As far as the C 1s spectrum is concerned, in addition to adventitious carbon, C–O chemical species are incorporated into the film composite.

In the case of PE-ALD AlO_*x*_, unlike the ALD counterpart, the underlying MoS_2_ film is slightly oxidized. However, the oxidation is to a smaller extent as compared with the PE-ALD HfO_*x*_ case. The Mo^6+^/Mo^4+^ ratio for the PE-ALD AlO_*x*_ case is 0.11, whereas it is 0.52 for the PE-ALD HfO_*x*_ case (see Table S2 for a quantitative analysis). In addition, both the Mo 3d and S 2p major peaks shift −1.0 eV, being another indication of MoS_2_ surface oxidation. Considering the C 1s spectrum, C–O and carboxyl chemical states are detected.

Upon the growth of ∼5 nm of HfO_*x*_ on MoS_2_, similar to what was observed earlier in Figure 2 for the growth of ∼2.6 nm of HfO_*x*_ at 100 °C, there is again evidence of MoS_2_ surface oxidation. In this case, the Mo^4+^ and the S 2p doublets shift −0.9 eV. In addition, the Mo^6+^/Mo^4+^ ratio increases to 0.52 (see Table S2 for the details). As the Mo^6+^ and Mo^4+^ doublet peaks are not clearly distinguishable in the graph above, this spectrum is provided separately in Figure S5. In the C 1s spectrum, apart from the C–C adventitious peak, two other chemical states (C–O and C(=O)–OH (carboxyl)) are detected at 287.1 and 289.4 eV, respectively. Compared with the bare MoS_2_, the integrated area under the carboxyl peak increases upon the growth of HfO_*x*_, suggesting that a moderate amount of carbon-related content is incorporated into PE-ALD grown HfO_*x*_.

These interface observations draw our attention to several notable points:

First of all, unlike H_2_O, the employment of O_2_ plasma reactant during the PE-ALD process of AlO_*x*_ and HfO_*x*_ oxidizes the MoS_2_ surface, which is expected to facilitate the growth of these dielectrics on MoS_2_.^24^ The O_2_ plasma duration for the HfO_*x*_ process is 8 s in each deposition cycle, whereas for the AlO_*x*_ it is 2 s (see the Supporting Information for the details of each process, Sections S.2.2 and S.2.3). The shorter plasma exposure time during the PE-ALD of AlO_*x*_ can partly explain the lower Mo^6+^ content (Table S2) compared with what is obtained from the PE-ALD of HfO_*x*_ on MoS_2_. The reader should note that for the PE-ALD of AlO_*x*_ and HfO_*x*_ films, standard recipes (which had been developed earlier) were simply used. This clarifies why processing conditions (e.g., O_2_ plasma duration) are different in these two processes.

The second point to highlight is that because H_2_O does not react strongly with MoS_2_, there is most likely a nucleation delay during the early ALD cycles of AlO_*x*_. Here, it is noted that all the dielectric layer thicknesses, as referred to in this study, were determined on control samples of SiO_2_/Si. The nucleation delay of ALD AlO_*x*_ on MoS_2_ could lead to a thickness deviation between what is deposited on MoS_2_ and that on SiO_2_/Si during the early cycles of the deposition.

Finally, for both ALD and PE-ALD processes, carbon impurities are incorporated into the dielectric film because of the low growth temperature of 100 °C and subsequently the incomplete ALD reactions.^59^

#### 3.2.3 Linking XPS Results to *I*–*V* Characterization

After these extensive XPS analyses, it becomes clear that the AlO_*x*_ films, processed by both the ALD and PE-ALD methods, contain a measurable amount of C–O and C(=O)–OH (carboxyl) species. The presence of these carbon-related compounds can be a plausible explanation for the observed *n*-type device performance during the *I–V* measurements.^47^ One may also wonder about the role of MoO_*x*_, which is known as a hole dopant for MoS_2_. It is worth pointing out that the detected concentration of MoO_*x*_ after both the ALD and PE-ALD AlO_*x*_ processes at 100 °C is not so significant. The Mo^6+^/Mo^4+^ ratios (see Table S2) are only 0.07 and 0.11 for the ALD and PE-ALD AlO_*x*_ cases, respectively. This is while the Mo^6+^/Mo^4+^ ratio is 0.52 for the reference case (HfO_*x*_-capped MoS_2_ FETs). Therefore, a substantial hole transfer into the MoS_2_*E*_v_ is less likely.

To summarize, although our ALD/PE-ALD AlO_*x*_ films are oxygen-rich, the MoS_2_ FETs capped with these dielectrics still show *n*-type performance, which can be the result of carbon impurities incorporated into the AlO_*x*_ films when deposited at low temperatures. Furthermore, the devices capped with PE-ALD AlO_*x*_ exhibit the most optimal characteristics, as they show higher *I*_ON_/*I*_OFF_ and lower *I*_OFF_ among the three studied cases. They also perform better than the ones capped with the ALD AlO_*x*_ in the ON-state regime, mostly due to the generally higher dielectric constant of PE-ALD AlO_*x*_ than the ALD counterpart.

### 3.3 AlO_*x*_/HfO_*x*_ Bilayer Capping

In conventional FETs, the growth of high-κ bilayers (AlO_*x*_ with HfO_*x*_ or ZrO_*x*_) is one strategy to increase the total gate dielectric capacitance and to further scale down the dielectric thickness without diminishing the gate electrostatic control over the channel.^66−68^ The complementary features of AlO_*x*_ and HfO_*x*_ (or ZrO_*x*_) (e.g., their dielectric constant, *E*_g_, etc.)^69^ make their bilayer stacks suitable options for gate dielectrics. As far as the MoS_2_ FETs are considered, in addition to the increased gate electrostatic control,^26^ the bilayer approach can better screen the CI scattering in MoS_2_.^34^ Therefore, it is expected that the employment of dielectric bilayers further improves the MoS_2_ device metrics. To verify this, stacks of AlO_*x*_ and HfO_*x*_ were examined on our fabricated devices. The AlO_*x*_ interlayer was grown by both ALD and PE-ALD methods and the HfO_*x*_ by PE-ALD, all at 100 °C. The deposition conditions were kept similar to those when these films were grown individually on the MoS_2_ FETs. The entire stack thickness was also retained at 30 nm while different thickness combinations were investigated.

#### 3.3.1 2.5/27.5 nm of AlO_*x*_/HfO_*x*_

##### *I*–*V* Characterization of 2.5/27.5 nm AlO_*x*_/HfO_*x*_ Bilayer Stack

**Figure 5:**
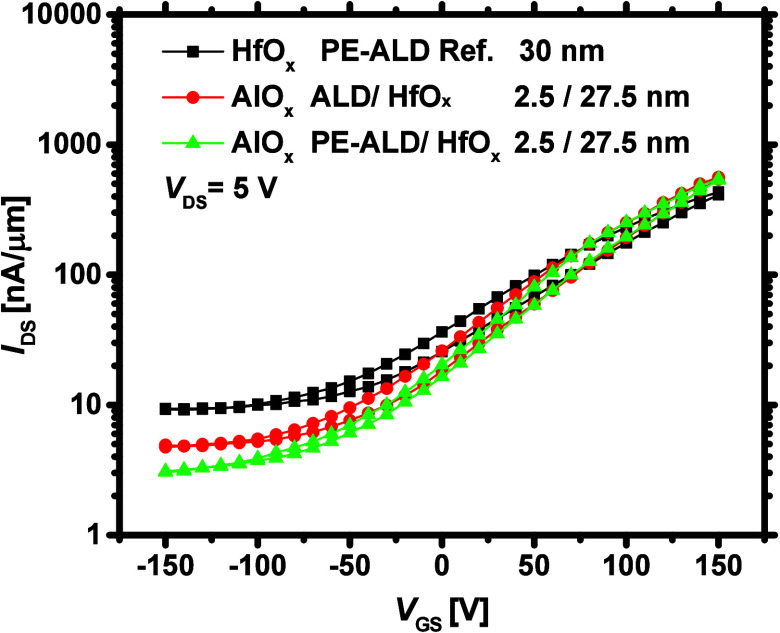
Transfer curves (in semilog scale) of the MoS_2_ FETs capped with bilayers of AlO_*x*_ and HfO_*x*_ (where AlO_*x*_ is grown by both ALD and PE-ALD processes).

Figure 5 shows the transfer characteristics of the MoS_2_ FETs in semilog scale, capped with 2.5/27.5 nm of AlO_*x*_/HfO_*x*_, where the AlO_*x*_ interlayer is grown by both ALD and PE-ALD. For the transfer curves in linear scale, see Figure S6. Compared with the reference, when the bilayer approach is followed, *V*_T_ shifts more positively (from ∼40 to ∼70 V, in forward sweep), and *I*_OFF_ reduces. These observations indicate that upon the growth of bilayer stacks with the oxygen-rich AlO_*x*_ interlayer, MoS_2_ is *n*-type doped to a lesser extent as compared to the reference. However, *I*_ON_ slightly improves, which can primarily be attributed to the more effective screening of MoS_2_ CIs (as scattering centers) when bilayer stacks are employed.^34^ In fact, the total dielectric constant of a bilayer stack can be higher than that of a single layer, as verified in previous studies where AlO_*x*_/HfO_*x*_ stack resulted in higher dielectric constant than HfO_*x*_.^69,70^

The average statistical data for both the ON- and OFF-states of these devices also show similar trends (Figure S6).

##### XPS Analysis of 2.5/2.5 nm AlO_*x*_/HfO_*x*_ Bilayer Stack

To evaluate the chemical states at the bilayer high-κ/MoS_2_ interface, thin bilayers must be used. The average XPS depth of analysis (using Al Kα radiation source (*hv* = 1486.6 eV)) is limited to the upper ∼10 nm of a stack. Sputter depth profiling to access a deeper lying interface is also not a preferred option because of the potential reduction of oxidic states upon the sputtering process. Therefore, the XPS analysis is performed on only 2.5/2.5 nm thick ALD AlO_*x*_/PE-ALD HfO_*x*_ stacks grown on the MoS_2_ films.

**Figure 6:**
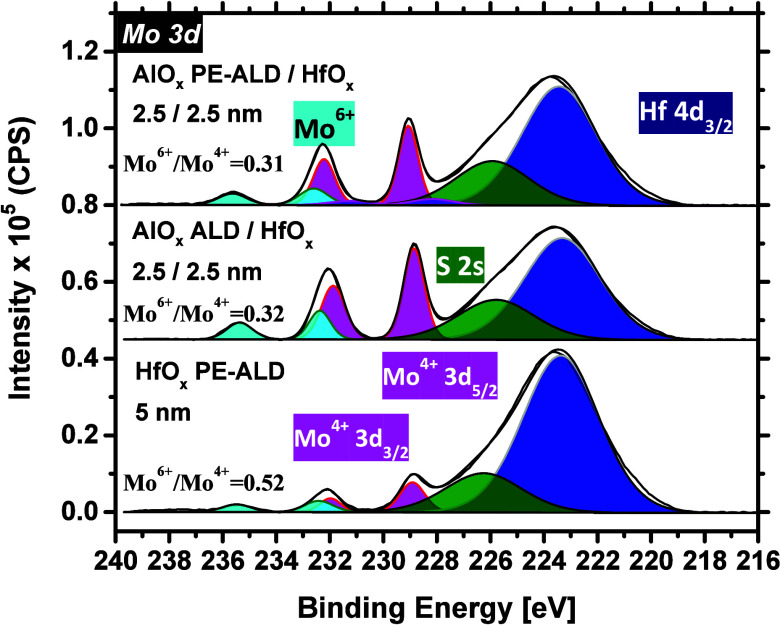
Mo 3d core level spectra after the growth of 2.5/2.5 nm AlO_*x*_/HfO_*x*_ films on MoS_2_ as compared with 5 nm pure HfO_*x*_ on MoS_2_.

Figure 6 illustrates the Mo 3d core level spectra of the studied cases. The S 2p and C 1s spectra are also provided in Figure S7. The data are compared to those of the ∼5 nm HfO_*x*_ deposited on MoS_2_. As can be seen from the plot, after the growth of both bilayer stacks, the Mo 3d major peak intensities do not drop as dramatically as upon the pure HfO_*x*_ growth, inferring that less dielectric (than expected) is grown on MoS_2_ with the bilayer approach. This is also evidenced from the higher intensity of the Si 2p spectrum for the bilayer cases (especially the ALD AlO_*x*_) than for the HfO_*x*_ counterpart (see Figure S7). As mentioned earlier, a nucleation delay likely occurs upon the growth of AlO_*x*_ films on MoS_2_ and during the initial deposition cycles, as H_2_O and O_2_ plasma used in the ALD and PE-ALD processes of AlO_*x*_ do not react so strongly with MoS_2_ (compared with the O_2_ plasma in the HfO_*x*_ process). This is the major cause of the higher Mo^4+^ peak intensity for the AlO_*x*_/HfO_*x*_ stack cases than for the single layer HfO_*x*_ counterpart.

The AlO_*x*_ and HfO_*x*_ process specifications are provided in Section S.2 of the Supporting Information for further clarifications.

Furthermore, the Mo^6+^/Mo^4+^ ratio for the HfO_*x*_ and the bilayer cases with ALD and PE-ALD AlO_*x*_ interlayers are 0.52, 0.32, and 0.31, respectively (see Table S3). In other words, a portion of the MoS_2_ surface is oxidized upon the 2.5/2.5 nm bilayer growth and irrespective of the AlO_*x*_ processing method. This is unlike the growth of 5 nm single layer ALD and PE-ALD AlO_*x*_ on MoS_2_, where the Mo^6+^/Mo^4+^ is only 0.07, 0.11 (pointed out previously in Table S2). Therefore, one can speculate that the 2.5 nm AlO_*x*_ is most likely not a closed layer, such that the HfO_*x*_ process can still significantly oxidize the MoS_2_ underneath (but evidently less than when only pure HfO_*x*_ is employed).

##### STEM Analysis of the Dielectric Growth on MoS_2_

To further elucidate the dielectric coverage on MoS_2_, the growth and microstructure of the high-κ/MoS_2_ stacks are evaluated by using top-view STEM imaging as well as EDX mapping analyses. Initially, the deposition of merely AlO_*x*_ films on MoS_2_ is discussed. Then, a study of the bilayers is provided.

**Figure 7:**
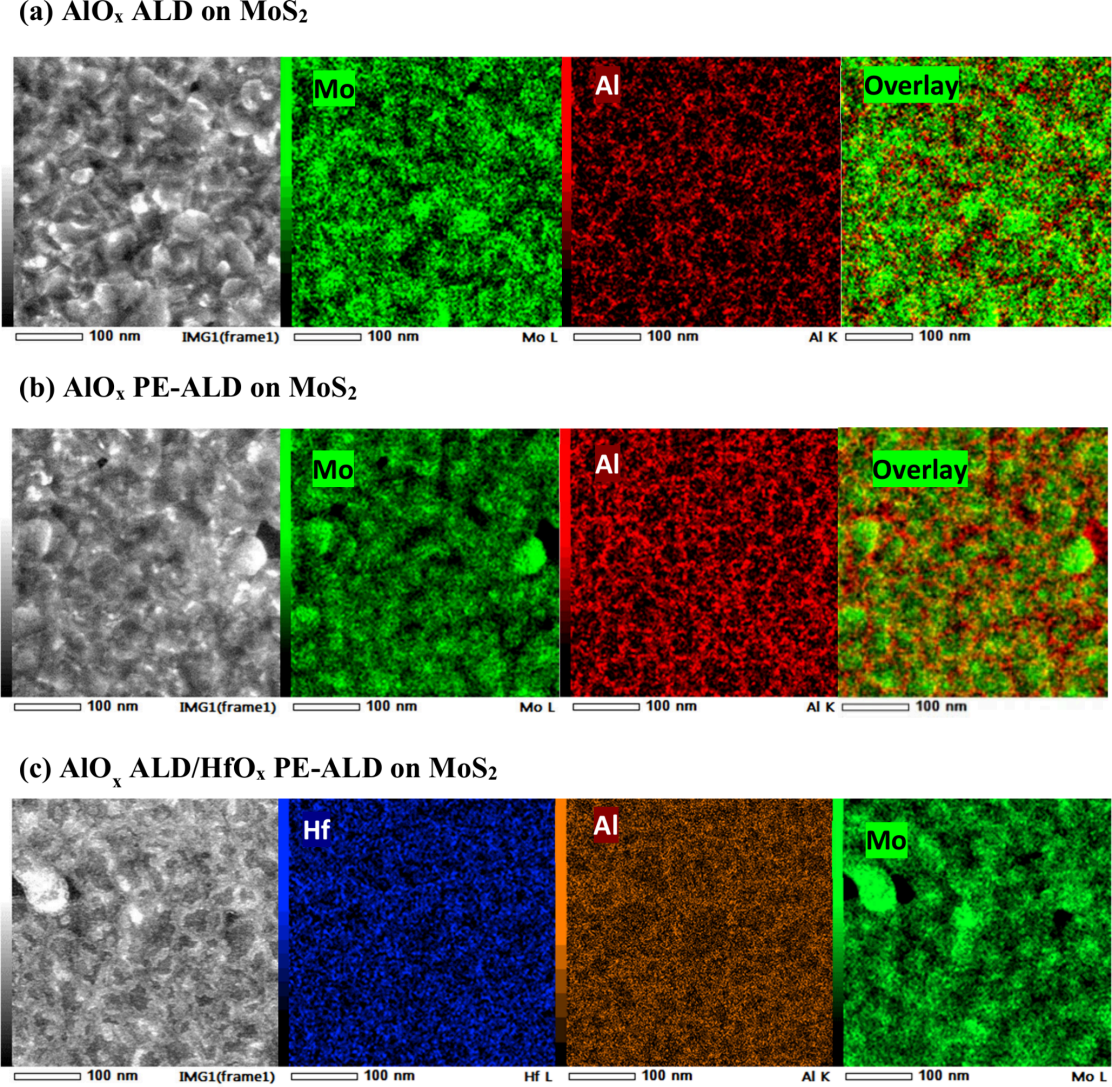
(a) HAADF-STEM image of 2.5 nm ALD AlO_*x*_ on MoS_2_ as well as the EDX elemental mappings of the Al, Mo, and their overlay image. (b) HAADF-STEM image of 2.5 nm PE-ALD AlO_*x*_ on MoS_2_ as well as the EDX elemental mappings of the Al, Mo, and their overlay image. (c) HAADF-STEM of 2.5/2.5 nm ALD AlO_*x*_/PE-ALD HfO_*x*_ on MoS_2_ as well as the EDX elemental mappings of Hf, Al, and Mo.

Figure 7a shows the high angle annular dark field (HAADF) mode STEM image of 2.5 nm ALD AlO_*x*_ on MoS_2_ as well as the EDX elemental mapping of Al and Mo and the color-coded overlays, respectively. As can be seen from the HAADF-STEM image, the MoS_2_ films are polycrystalline with a grain size of on average ∼70 nm.^55^ The GBs on MoS_2_ are known to be more reactive than the basal planes, as they are composed of defects that are arranged linearly. Therefore, AlO_*x*_ tends to nucleate predominantly at the GBs rather than on the basal planes, as evidenced from the mapping. This leads to a discontinuous layer of ALD AlO_*x*_ on MoS_2_ after ∼2.5 nm of growth, which has also been observed previously.^53,54^Figure 7b displays the EDX overlay mapping of ∼2.5 nm PE-ALD AlO_*x*_ on MoS_2_. Overall, the nucleation density of PE-ALD AlO_*x*_ on MoS_2_ is more than when it is grown by ALD on MoS_2_. This is mainly attributed to the use of O_2_ plasma during the PE-ALD process and its reactive components that promote the precursor chemical adsorption on both the MoS_2_ GBs and its basal planes. However, a completely closed layer of AlO_*x*_ is not yet achieved, even with the use of PE-ALD.

After realizing that 2.5 nm of AlO_*x*_ with neither of the deposition methods results in a closed layer of AlO_*x*_ on MoS_2_, we evaluated the bilayer high-κ growth on MoS_2_. Figure 7c provides the STEM-HAADF image of a 2.5/2.5 nm thick AlO_*x*_/HfO_*x*_ stack on MoS_2_ as well as the elemental mappings of Hf, Al, and Mo. Here, the AlO_*x*_ interlayer has only been grown by the ALD method (and HfO_*x*_ by the PE-ALD). As can be seen, although the AlO_*x*_ interlayer mostly decorates the MoS_2_ GBs, HfO_*x*_ is homogeneously deposited almost everywhere. Higher resolution HAADF-STEM images are also provided in Figure S8 for further visualization. The closed nature of the HfO_*x*_ layer on MoS_2_ strongly implies that the XPS results obtained from the 2.5/2.5 nm AlO_*x*_/HfO_*x*_ stack are also representatives for stacks with thicker HfO_*x*_ overlayers (e.g., 27.5 nm), which were used for capping the MoS_2_ FETs.

##### Linking All the Characterizations

When correlating the STEM images and the EDX mapping analysis jointly with the XPS measurements, one can comprehend that a 2.5 nm AlO_*x*_ interlayer (grown by ALD or PE-ALD) is not sufficient for attaining a closed AlO_*x*_ dielectric film on MoS_2_. Therefore, when the bilayer approach is followed, HfO_*x*_ can still reach the underlying MoS_2_ and react with the MoS_2_ surface. This explains the increase in the Mo^6+^ content upon the growth of both bilayer cases on MoS_2_ (compared with when only single layers of AlO_*x*_ are grown).

However, the intensity of the Mo^6+^ chemical state for the bilayer cases is not higher than what is detected for the pure HfO_*x*_ case. The Mo^6+^/Mo^4+^ ratio for the bilayer cases is ∼0.3 and 0.5 for the HfO_*x*_ case (Table S3). In other words, for bilayer cases, the concentration of MoO_*x*_ on the MoS_2_ surface is less than that for the pure HfO_*x*_ case. In such a situation, one would expect a relative increase in the *n*-type doping of the devices capped with AlO_*x*_/HfO_*x*_ stacks, since MoO_*x*_ is known as a hole dopant for MoS_2_, and its reduced concentration denotes less *p*-type doping of the devices. However, the reverse behavior is observed. *V*_T_ shifts positively, and *I*_OFF_ reduces, as compared with the reference device (Figure 5a,b). This implies a relative *n*-type doping reduction (relative *p*-type doping increase) upon capping the MoS_2_ FETs with dielectric bilayers. As previously pointed out, our AlO_*x*_ films are oxygen-rich (grown with both ALD and PE-ALD processes). Furthermore, it has been shown that the excess oxygen states of the AlO_*x*_ are energetically located in the vicinity of MoS_2_*E*_v_ and can dope the MoS_2_ to *p*-type.^7,44^ This could be a plausible explanation for relatively lower *n*-type doping (relatively higher *p*-type doping) of our devices, despite the lower concentration of MoO_*x*_ on MoS_2_ upon the growth of bilayers.

#### 3.3.2 5/25 nm AlO_*x*_/HfO_*x*_

The next logic step is to increase the AlO_*x*_ interlayer thickness to 5 nm while maintaining the entire stack thickness at 30 nm.

##### *I*–*V* Characterization of the 5/25 nm AlO_*x*_/HfO_*x*_ Bilayer Stack

**Figure 8:**
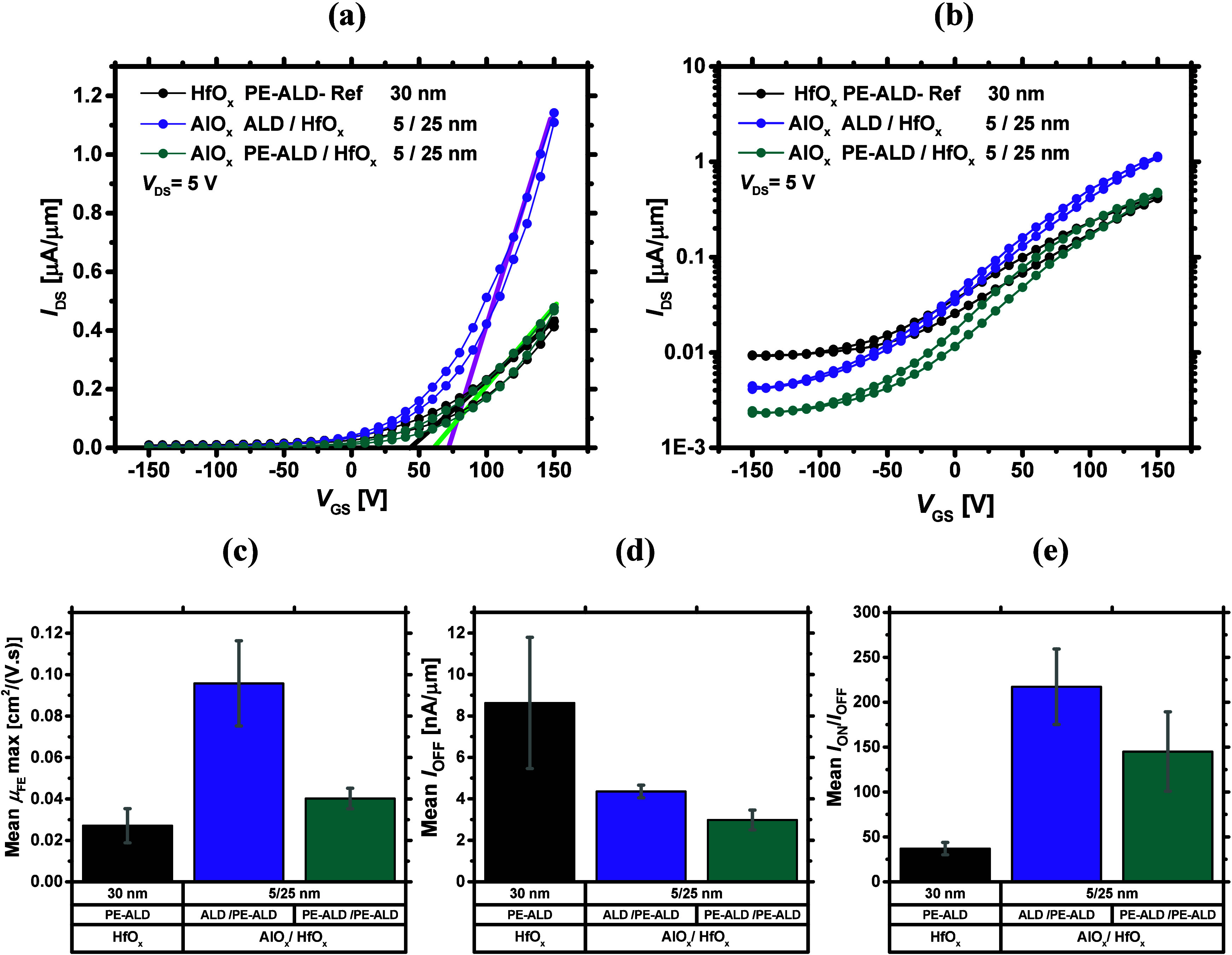
Transfer curves of the fabricated MoS_2_ FETs, capped with bilayers of 5/25 nm AlO_*x*_/HfO_*x*_ in (a) linear and (b) semilog scales. The straight lines in (a) and their interception with the horizontal axis correspond to the approximate values of *V*_T_ (c–e) are the average statistical data of the maximum μ_FE_, *I*_OFF_, and *I*_ON_/*I*_OFF_, respectively.

Figures 8a and 8b compare the transfer characteristics of the MoS_2_ FETs capped with 5/25 nm of bilayers with the reference, on linear and semilog scales. As can be seen, the growth of the 5 nm AlO_*x*_ interlayer shifts *V*_T_ to more positive voltages and reduces *I*_OFF_, relative to the reference, again implying that the underlying MoS_2_ has become less *n*-type doped. The approximate *V*_T_ values are marked by straight lines in Figure 8a. Upon the ALD growth of a 5 nm AlO_*x*_ interlayer, *I*_ON_ improves more than 2-fold and reaches 1.2 μA/μm. In addition, the maximum μ_FE_ increases to 0.1 cm^2^/(V·s) on average. *I*_OFF_ halves and the *I*_ON_/*I*_OFF_ ratio becomes the highest (∼250) for the ALD AlO_*x*_ interlayer case, as provided in Figure 8c–e and statistically. The ON-state enhancement is most likely the result of a higher dielectric constant for this specific bilayer combination,^69,70^ such that the MoS_2_ CIs can be more effectively screened. This leads to the best achieved electrical performance to date for our ALD-based MoS_2_ FETs. Considering the PE-ALD AlO_*x*_/HfO_*x*_ case, although *I*_OFF_ is the lowest, *I*_ON_ improves only slightly (see Figure S9), causing the maximum μ_FE_ only as high as 0.04 cm^2^/(V·s) and the *I*_ON_/*I*_OFF_ ratio of ∼150 (Figures 8c and 8e, respectively). For elucidating these observations, the chemistry involved at the high-κ/MoS_2_ interface is again investigated by using XPS.

##### XPS Analysis of the 5/2.5 nm AlO_*x*_/HfO_*x*_ Bilayer Stack

**Figure 9:**
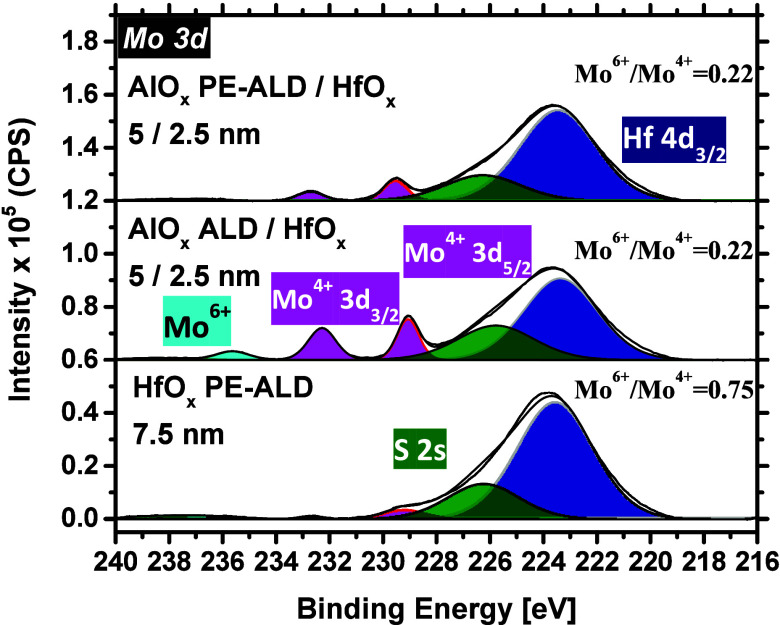
Mo 3d spectra after the growth of 7.5 nm HfO_*x*_ (PE-ALD) and 5/2.5 nm AlO_*x*_/HfO_*x*_ where the interlayer is grown by both the ALD and PE-ALD methods.

The XPS analysis is performed after the growth of ∼5/2.5 nm AlO_*x*_/HfO_*x*_ combinations on bare MoS_2_ films. As can be seen in Figure 9, upon the growth of these bilayer stacks, the Mo 3d major peaks do not drop as notably when the pure PE-ALD HfO_*x*_ is grown. As discussed before, unlike the PE-ALD of HfO_*x*_, our AlO_*x*_ films experience a nucleation delay on MoS_2_ during the initial deposition cycles of both ALD and PE-ALD processes. This results in some deviations between the expected and actual AlO_*x*_ thicknesses on MoS_2_, subsequently leading to a lower drop in the intensity of Mo^4+^ doublet peaks. Evaluating the substrate Si 2p core level spectrum (provided in Figure S10) also confirms that less AlO_*x*_ is grown on MoS_2_, as compared with the case of HfO_*x*_. In fact, when a thicker dielectric is grown on MoS_2_, the Si 2p peak intensity fades out due to the limitations in the XPS average depth of analysis. The total counts per second (CPS) area under the Mo^4+^ and Mo^6+^ doublet peaks are evaluated as well (see Table S4 for further details). For the pure HfO_*x*_ case, the Mo^6+^/Mo^4+^ ratio is 0.75, whereas for both bilayer cases, it does not exceed 0.22, suggesting that the addition of a 5 nm AlO_*x*_ interlayer better protects the MoS_2_ surface from being oxidized upon the HfO_*x*_ deposition. Here, it is remembered that the Mo^6+^/Mo^4+^ ratio is ∼0.3 when a 2.5/2.5 nm AlO_*x*_/HfO_*x*_ stack is grown on MoS_2_ (Table S3).

##### Linking XPS Data to *I*–*V*

Linking the XPS analyses to the *I–V* measurements, similar trends can be observed as to when 2.5/2.5 nm bilayer stacks are grown on MoS_2_. Again, the Mo^6+^/Mo^4+^ ratios for the bilayer cases are less than that of HfO_*x*_ (evidenced in the XPS), implying that the MoS_2_ surface is less oxidized to MoO_*x*_. Therefore, the observed *V*_T_ positive shift and the *I*_OFF_ reduction in the devices capped with 5/25 nm bilayer dielectrics (relative to the reference) cannot be attributed to the hole injection from the MoO_*x*_, and they are mainly due to the oxygen-rich states of AlO_*x*_ that reside in close proximity of the MoS_2_*E*_v_.

#### 3.3.3 10/20 nm AlO_*x*_/HfO_*x*_

**Figure 10:**
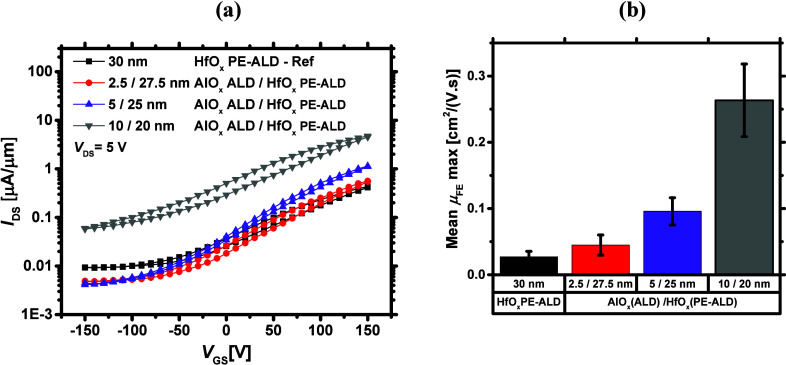
(a) Transfer curves of the fabricated MoS_2_ FETs capped with bilayer dielectrics of different AlO_*x*_ interlayer thicknesses. The AlO_*x*_ interlayer is grown by ALD. (b) Mean maximum μ_FE_ and its dependence on the AlO_*x*_ interlayer thickness.

It is also worth evaluating the 10/20 nm dielectric stack on the fabricated MoS_2_ FETs. Figure 10a shows the transfer curves of the devices capped with the ALD AlO_*x*_/PE-ALD HfO_*x*_ of 2.5/27.5, 5/25, and 10/20 nm combinations and the reference. Here, we only tried the ALD of AlO_*x*_ (and not the PE-ALD of AlO_*x*_) as we deduced that the ALD of AlO_*x*_ best protects the MoS_2_ surface from being oxidized to MoO_*x*_. As can be seen from Figure 10a, by raising the AlO_*x*_ interlayer thickness to 10 nm, *I*_ON_ improves nearly 1 order of magnitude and reaches 4.7 μA/μm, as compared with the reference. A similar trend can also be observed for the mean maximum μ_FE_, which is shown in Figure 10b. The statistical data for other device metrics are also provided in Figure S11. Despite the improvements in the device ON-state performance, *V*_T_ shifts negatively, and *I*_OFF_ degrades dramatically for the 10/20 nm bilayer case, implying that our devices become degenerately *n*-type doped upon the growth of 10/20 nm dielectric stack. As a result of this excess *n*-type doping, the back-gate bias fails to properly deplete the channel in the OFF-state regime. This suggests that when a 10/20 nm dielectric stack is grown on the MoS_2_ FETs, a surface channel is formed.^31^ In fact, only the topmost layers of the MoS_2_ are electrically active, which leads to a weak back-gate electrostatic control over the channel.

From the material point of view, growing 10 nm of the ALD AlO_*x*_ interlayer uniformly covers the underlying MoS_2_. This is shown in the STEM top view images provided in Figure S12.

##### Stability and Repeatability of the Optimal Devices

**Figure 11:**
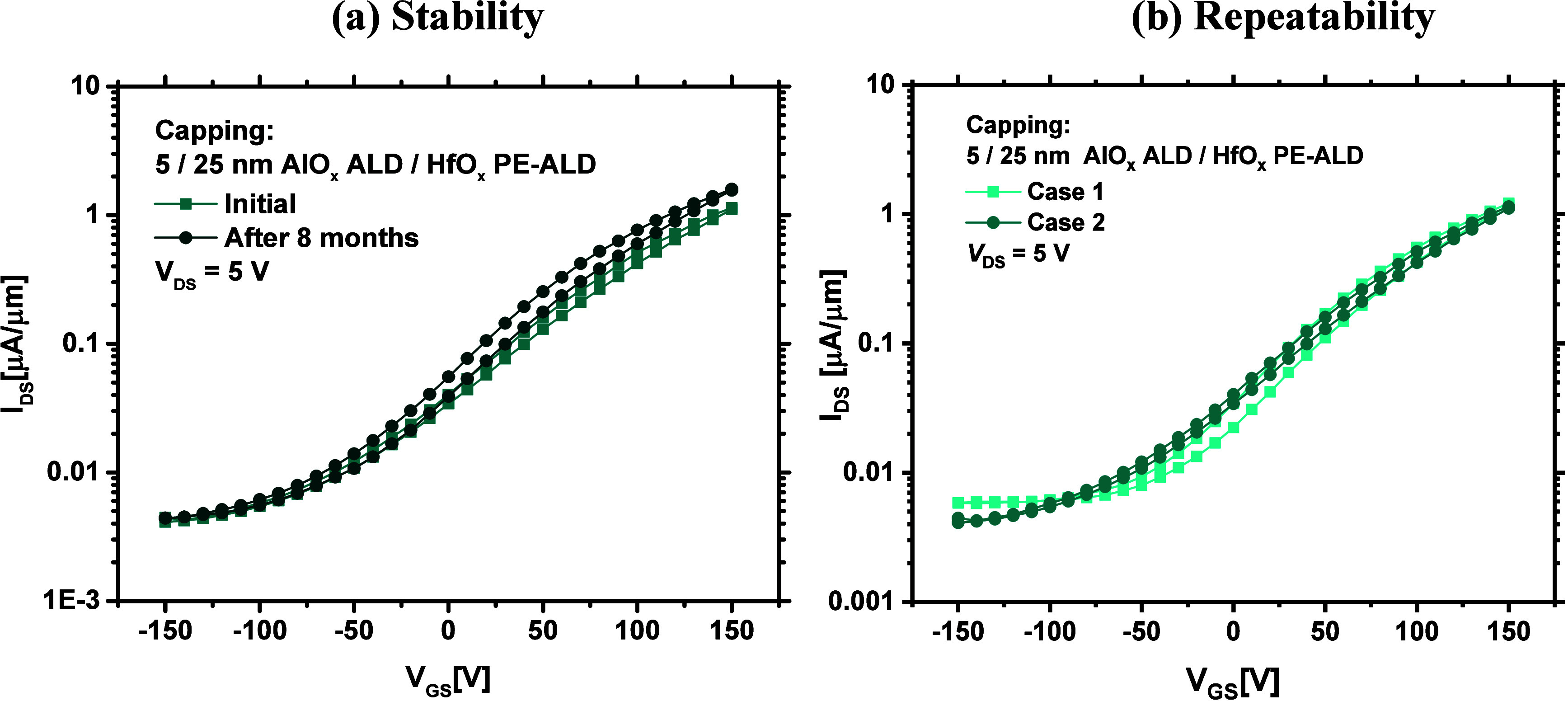
(a) Initial transfer characteristics of a fabricated MoS_2_ FET, capped with 5/25 nm of ALD AlO_*x*_/PE-ALD HfO_*x*_ dielectrics, and after eight months of being remeasured. (b) Transfer characteristics of two devices made with same processing conditions on two different wafers.

Based on the above discussions, among all the studied cases, the optimal device performance is obtained when 5/25 nm of ALD AlO_*x*_/PE-ALD HfO_*x*_ is grown on the fabricated MoS_2_ FETs. It is also worthwhile evaluating the stability and repeatability of the data obtained from such devices. Figure 11a compares the *I–V* performance of the same device after eight months of being initially measured. As evidenced, the device metrics are persistent over a long period of time, indicating a high degree of device stability when capped with such a dielectric stack. The *I–V* performance of a device fabricated few months after, on another wafer and with similar processing conditions is also shown in Figure 11b. The data verify that the obtained results for devices capped with bilayers of such a thickness combination (5/25 nm) are repeatable.

## 4 Conclusions

To conclude, this work extensively investigates the integration of high-κ dielectrics with ALD-based MoS_2_ FETs by means of electrical, surface-chemical, and material characterization techniques.

The electrical characterization data show that the employment of high-κ dielectrics improves the overall device metrics in ALD-based MoS_2_ FETs. While other studies have associated such improvements mainly with the effective screening of MoS_2_ charged impurities (CIs) by a high-κ dielectric as well as the dielectric doping effect, we show that three additional parameters related to the processing of the dielectrics can simultaneously govern the MoS_2_ FET electrical performance and control its doping. These are the stoichiometry of the dielectrics, the carbon impurity content in the dielectrics, and the oxidation degree of the MoS_2_ top surface upon the dielectric growth.

Despite the fact that our AlO_*x*_ films are oxygen-rich, which typically leads to *p*-type doping in MoS_2_ FETs, an overall *n*-type performance is observed in the devices capped with these dielectrics. Surface chemical analysis reveals that carbon-related species are incorporated into the AlO_*x*_ films when grown at 100 °C, being one plausible explanation of the overall observed *n*-type characteristics in MoS_2_ FETs capped with oxygen-rich AlO_*x*_. Among the single layer dielectrics studied, the PE-ALD AlO_*x*_ films exhibit the most optimal device electrical performance, as the resulting devices exhibit a high *ON*-state current while maintaining low OFF-state characteristics.

Surface-chemical analyses further show that the PE-ALD of HfO_*x*_ and AlO_*x*_ dielectrics oxidize the MoS_2_ surface to MoO_*x*_, whereas the (thermal) ALD AlO_*x*_ leaves the underlying MoS_2_ surface almost intact. Therefore, if a layer of (thermal) ALD AlO_*x*_ is employed between MoS_2_ and PE-ALD HfO_*x*_, device metrics improve further. Among the bilayer cases, the 5/25 nm (thermal) ALD AlO_*x*_/PE-ALD HfO_*x*_ dielectric stack leads to the most optimal device electrical characteristics. We relate this to the overall higher dielectric constant of the bilayer stack as well as a fair protection of the MoS_2_ surface against oxidation upon the growth of HfO_*x*_. The MoS_2_ FETs capped with this bilayer combination also show a high degree of stability over time with repeatable results.

Our findings and the connections we make between the surface-chemical, material, and electrical analyses provide guidelines to further optimize and improve the properties of synthetic-based MoS_2_ FETs of polycrystalline MoS_2_ nature.

## Supporting Information Available

MoS_2_ FET optical microscopy top-view and cross-sectional schematics; process specifications for ALD and PE-ALD of AlO_*x*_ and HfO_*x*_; HfO_*x*_ capping layer on MoS_2_ (XPS and Raman data analyses); AlO_*x*_ capping layer on MoS_2_ (MoS_2_ FET device metrics and XPS data analysis); bilayer AlO_*x*_/HfO_*x*_ capping layer on MoS_2_ (MoS_2_ FET device metrics, XPS analyses, HR-STEM images); references (PDF)
