# Supplementary material for: Influence of High-κ Dielectrics Integration on ALD-Based MoS2 Field-Effect Transistor Performance
Source: ACS Appl Nano Mater. 2024 Aug 12;7(16):18786–800. doi: 10.1021/acsanm.4c02214 (PMC11348321; doi:10.1021/acsanm.4c02214)
Supplement: Supplementary file 1 — an4c02214_si_001.pdf [file an4c02214_si_001.pdf]

Supporting Information

Influence of High-κ Dielectrics Integration on ALD-Based MoS_2 Field-Effect Transistor Performance

Reyhaneh Mahlouji [1], *Yue Zhang* [2], *Marcel A. Verheijen* [1,3], *Saurabh Karwal* [5], *Jan P. Hofmann* [2,4], *Wilhelmus. M.M. Kessels* [1], *Ageeth A. Bol** [1,6]

[1]Department of Applied Physics, Eindhoven University of Technology, P.O. Box 513, 5600 MB Eindhoven, The Netherlands

[2]Laboratory of Inorganic Materials and Catalysis, Department of Chemical Engineering and Chemistry, Eindhoven University of Technology, P.O. Box 513, 5600 MB Eindhoven, The Netherlands

[3] Eurofins Materials Science, High Tech Campus 11, 5656 AE Eindhoven, The Netherlands

[4]Surface Science Laboratory, Department of Materials and Earth Sciences, Technical University of Darmstadt, Otto-Berndt-Strasse 3, 64287 Darmstadt, Germany

[5]Netherlands Organization for Applied Scientific Research (TNO), 2628 CK Delft, Netherlands

[6]Department of Chemistry, University of Michigan, 930 N. University Ave., Ann Arbor, MI 48109, USA

Corresponding Author: a.a.bol@tue.nl

S.1 MoS_2 FET Optical Microscopy Top-View and Cross-Sectional Schematics

Figure S1(a) and (b) show the top view microscopic image and the schematic cross-section of the fabricated MoS_2 devices capped with a layer of high-κ on top, respectively.

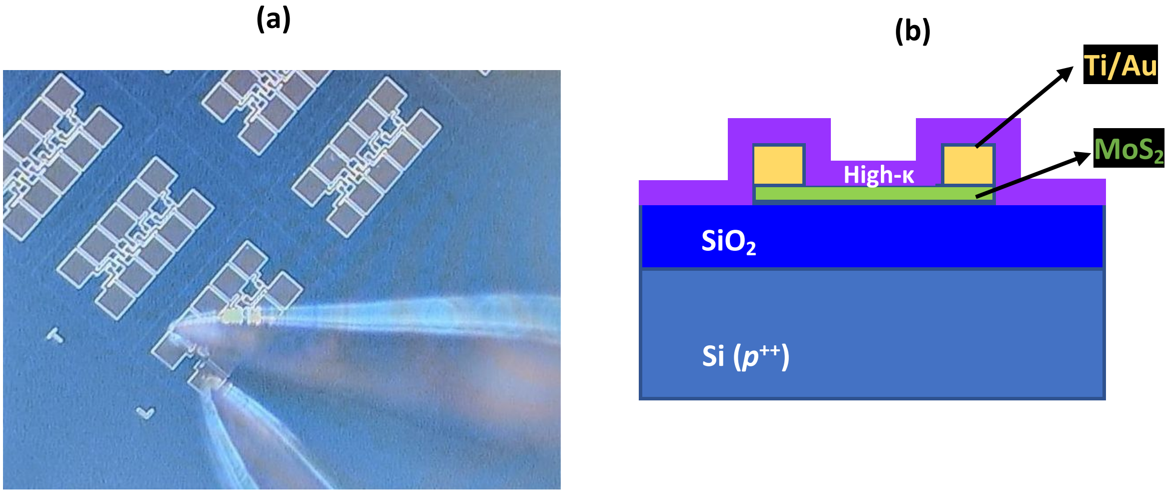

Figure S1. (a) Microscopic top view image of the fabricated devices, under measurement with probe tips. The visible patterns are the Ti/Au contact pads, (b) cross-sectional schematics of the fabricated MoS_2 devices.

S.2 Process Specifications for ALD and PE-ALD of AlO_x and HfO_x

S.2.1 PE-ALD of HfO_x

HfO_x films were grown on MoS_2 by using PE-ALD. HyALD and O_2 plasma were employed as the precursor and the oxygen agent, respectively. During the first half cycle, HyALD precursor was delivered into the reaction chamber with a 100 sccm of Ar bubbling flow. The dosing time was 3 s at a pressure of 200 mTorr, followed by a precursor purge step of 2 s with an Ar flow of 100 sccm. During the next half cycle, O_2 plasma was introduced for 8 s, with a flow rate of 100 sccm, a forward power of 400 W and at a pressure of 15 mTorr. The cycle was completed by a plasma purge step using 50 sccm of Ar for 3 s.

During both purge steps, the pressure was adjusted with an automatic pressure-controlled (APC) valve, to ensure that the lowest possible pressure in the chamber was achieved. Further details of the HfO_x synthesis process and film specifications were published previously by Sharma et al.[1]

S.2.2 ALD of AlO_x

During the ALD of AlO_x , trimethylaluminum (TMA, $Al(CH_3)_3$) vapor and H_2O were used as the reactants. Throughout the whole process, a constant flow of Ar was introduced with a rate of 100 sccm, and the chamber pressure was adjusted with an APC valve. When the APC valve was fully open (closed), the chamber pressure was the lowest (highest). The dosing time for the TMA precursor was 30 ms, and the subsequent Ar purging was 2 s long. In both steps, the APC was fully open. Next, H_2O was dosed into the chamber for 100 ms, followed by a reaction step of 1 s. The APC valve was fully closed for these two steps. Finally, the chamber and the manifolds were purged with an Ar flow for 3 s and 1 s respectively, meanwhile the APC valve was fully open.

S.2.3 PE-ALD of AlO_x

For the PE-ALD of AlO_x, TMA and O_2 plasma were used, and the pressure was adjusted with the APC unit. Throughout the growth process, a 100 sccm of Ar and 50 sccm of O_2 were continuously used, as O_2 does not react with TMA under the employed conditions. The TMA dosing time was 20 ms, and its purging duration was 1.5 s (both with a fully open APC). During the reaction step, an O_2 plasma was initiated into the chamber for 2 s, with a forward power of 200 W and at the highest achieved pressure (a fully closed APC). The subsequent plasma purge step was 0.5 s with a fully open APC.

S.3 HfO_x Capping Layer

S.3.1 C 1s Core Level Spectra

Figure S2, provides the C 1s spectrum before and after the growth of ~2.5 nm HfO_x on MoS_2 at different deposition temperatures. As can be seen from the plot, C(=O)-OH (carboxyl) species are present after the growth of HfO_x at 100 °C. This indicates that carbon impurities are incorporating into the HfO_x films at low deposition temperatures, and they can contribute to doping the MoS_2 devices to *n*-type[2] parallel to the HfO_x oxygen vacancies. The latter is already discussed in the main text. However, by elevating the deposition temperature to 200 °C and 300 °C, the FWHM of carboxyl species reduces, suggesting that C-content has less influential role in *n*-type doping of MoS_2.

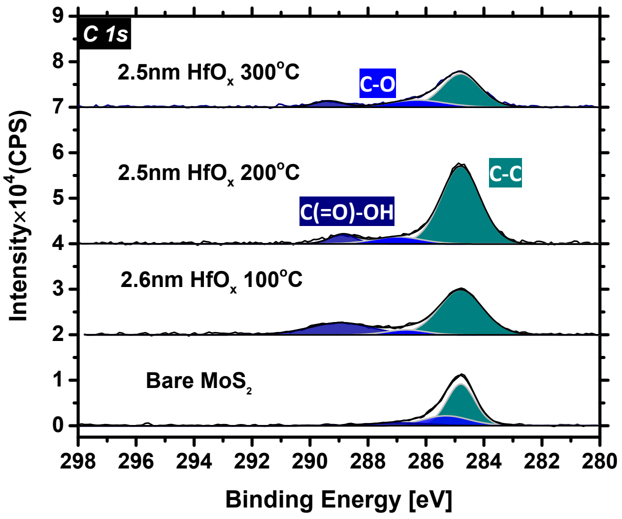

Figure S2 The C 1s core level spectra before and after the growth of HfO_x on MoS_2 at various deposition temperatures.

S.3.2 XPS Quantitative Details

To have an estimation on the degree of MoS_2 oxidation upon the growth of HfO_x at various temperatures, Mo^{6+}/Mo^{4+} ratio was determined from the integrated area under the Mo^{4+} and Mo^{6+} doublets. **Table S1** shows the obtained values for the Mo^{4+} and Mo^{6+} doublet peaks (counts per seconds (CPS)). In addition to the Mo^{4+} and Mo^{6+} values, the Mo $3d_{5/2}$ and S $2p_{3/2}$ peak positions as well as their relative binding energy (BE) shifts, with respect to bare the MoS_2 case, are provided in this table.

Table S1. The integrated area under the Mo^{4+} and Mo^{6+} peaks and their ratio, as well as the Mo $3d_{5/2}$ and S $2p_{3/2}$ peak positions and their BE shifts (with respect to the bare MoS_2 case) after the growth of 2.5 nm HfO_x at various deposition temperatures.

Sample	Area CPS Mo^{4+}	Area CPS Mo^{6+}	$\frac{Mo^{6+}}{Mo^{4+}}$	Mo 3d $_{5/2}$ peak [eV]	Mo 3d BE shifts [eV]	S 2p $_{3/2}$ peak [eV]	S 2p BE shifts [eV]
**Bare MoS_2**	132727	30031	0.22	229.8	0	162.6	0
**HfO_x 100°C/ MoS_2**	46593	20790	0.44	228.8	-1.0	161.7	-0.9
**HfO_x 200 °C/ MoS_2**	25739	34852	1.35	228.5	-1.3	161.4	-1.2
**HfO_x 300 °C/ MoS_2**	35156	45207	1.28	228.7	-1.1	161.5	-1.1

S.3.3 MoS_2 Raman Analysis

To verify that MoS_2 is structurally intact upon the growth of HfO_x on MoS_2, Raman analysis was employed. The set-up used for this purpose was Renishaw InVia confocal Raman microscopy, which was equipped with a 514 nm laser, an integrated switchable grating with 600 or 1800 lines/mm and a charge-coupled device (CCD) detector. During the Raman scans, 5 accumulations with an acquisition time of 10 s were taken, using a (laser power of <0.2 mW focused on a ~1 μm region). **Figure S3** shows the obtained data before and after the growth of 30 nm HfO_x films at various deposition temperatures. As can be seen, the characteristic MoS_2 Raman modes (A_{1g} and E^1_{2g} peaks) are yet present after the growth of HfO_x, irrespective of the deposition temperature, providing no significant evidence that the growth of HfO_x damages the entire underlying MoS_2.

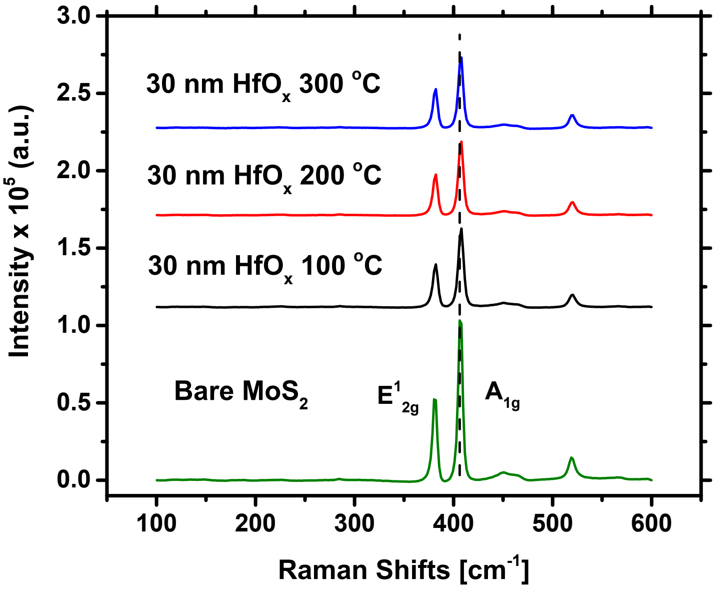

Figure S3. Raman analysis before and after the growth of 30 nm HfO_x films at various deposition temperatures.

S.4 AlO_x Capping Layer

S.4.1 Statistical Evaluation of Device Metrics

Figure S4(a), (b), (c) show the average statistical data for $I_{ON,}$ maximum μ_{FE} mobility, and I_{OFF} of the MoS_2 FETs, respectively, capped with ALD and PE-ALD of AlO_x as well as PE-ALD of HfO_x.

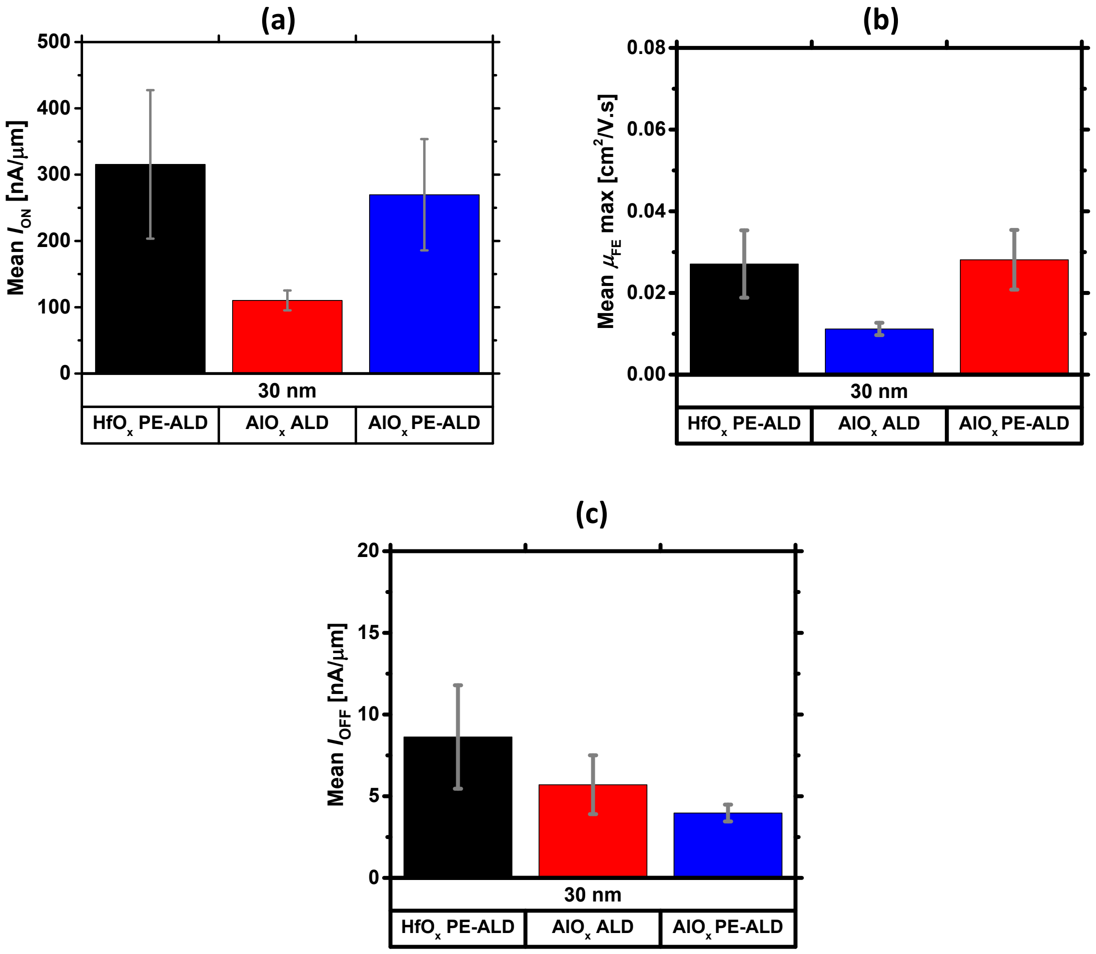

Figure S4. Average data for (a) I_{ON}, (b) the maximum μ_{FE} and (c) I_{OFF} upon ALD and PE-ALD of AlO_x as well as PE-ALD of HfO_x on the fabricated MoS_2 FETs.

S.4.2 Mo 3d Spectrum After the Growth of 5 nm HfO_x on MoS_2

Figure S5 shows the Mo3d spectrum after the growth of ~5 nm HfO_x at 100 °C, which is considered as our reference case. Here, the plot is provided separately for better visualization.

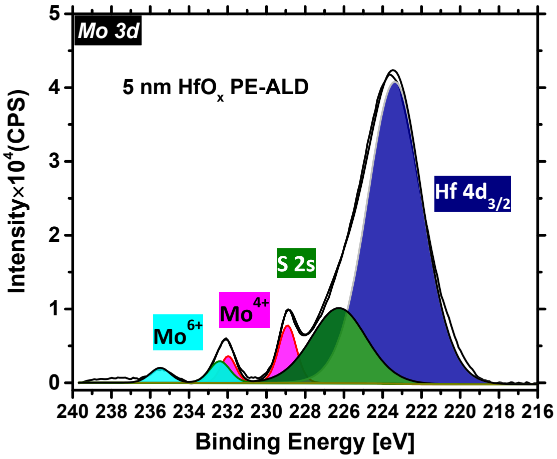

Figure S5 The Mo 3d spectrum after the growth of ~5 nm HfO_x on MoS_2 at 100 °C.

S.4.3 XPS Quantitative Details

Table S2 provides the details of the Mo^{6+} and Mo^{4+} chemical states, the Mo $3d_{5/2}$ and S $2p_{3/2}$ peak positions as well as their relative BE shifts, after the growth of 5 nm HfO_x and AlO_x dielectrics on MoS_2.

Table S2 CPS area under the Mo^{4+} and Mo^{6+} doublet peaks, their ratio, the Mo $3d_{5/2}$ and S $2p_{3/2}$ peak positions and their BE shifts after the growth of 5 nm of HfO_x and AlO_x .

MoS_2 Capping	Area CPS Mo^{4+}	Area CPS Mo^{6+}	$\frac{Mo^{6+}}{Mo^{4+}}$	Mo 3d $_{5/2}$ peak [eV]	Mo 3d BE shifts [eV]	S 2p $_{3/2}$ peak [eV]	S 2p BE shifts [eV]
**None**	132727	30031	0.22	229.8	0	162.6	0
**5 nm HfO_x PE-ALD**	12786	6710	0.52	228.9	-0.9	161.7	-0.9
**5 nm AlO_x PE-ALD**	58216	6550	0.11	228.8	-1.0	161.6	-1.0
**5 nm AlO_x ALD**	109726	8395	0.07	229.9	+0.1	162.7	+0.1

S.5 Bilayer AlO_x/HfO_x Capping Layer

S.5.1 The MoS_2 FET Device Metrics Capped with 2.5/27.5 nm AlO_x/HfO_x

Figure S6(a), (b), (c), (d) and (e) illustrate linear transfer curve, average max μ_{FE}, I_{ON}, I_{OFF} and I_{ON}/I_{OFF} data of the MoS_2 FETs, respectively, capped with 2.5/27.5 nm of AlO_x /HfO_x bilayers.

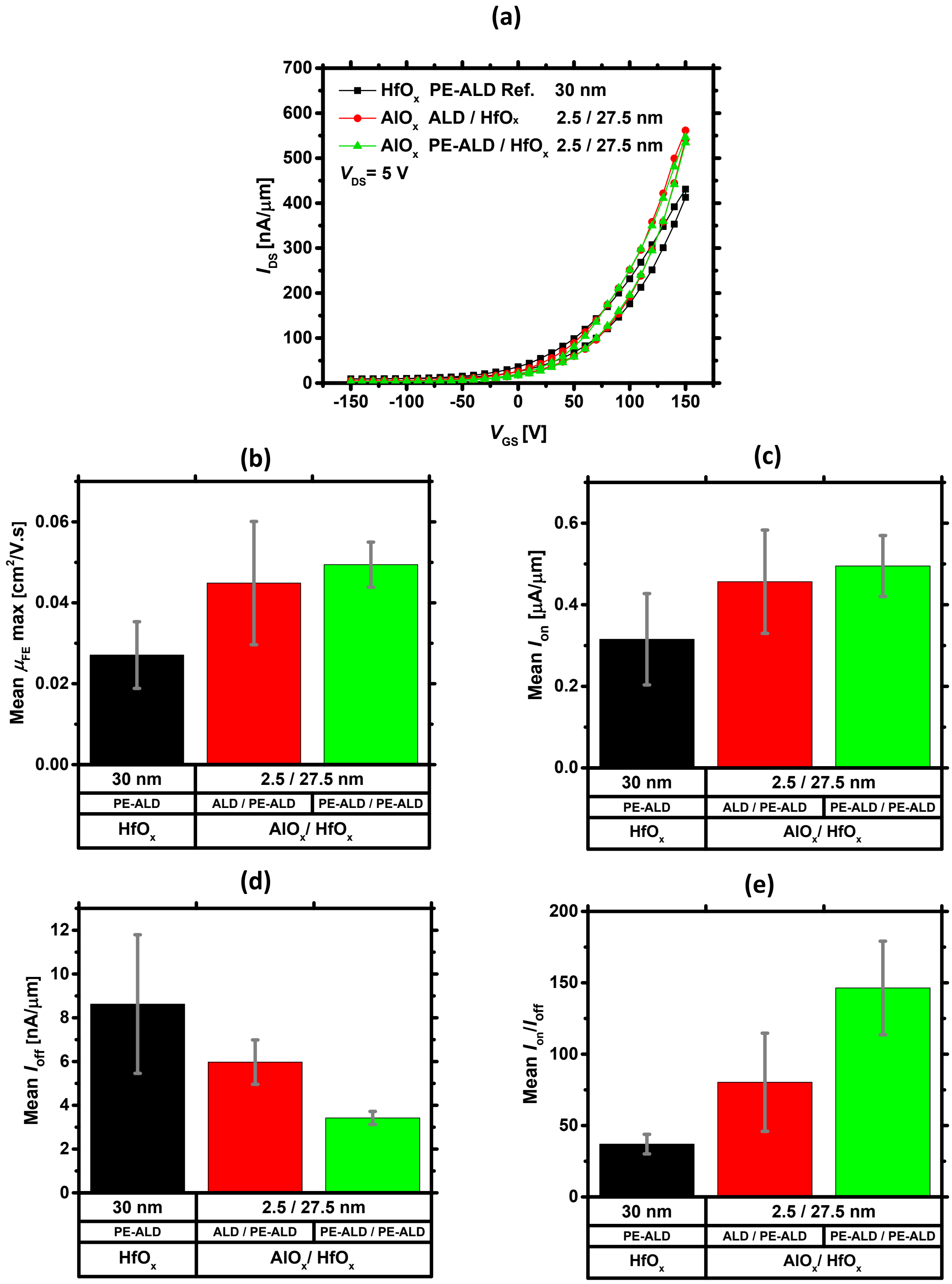

Figure S6. MoS_2 FET (a) linear transfer curve, (b) average maximum μ_{FE}, (c) average I_{ON}, (d) average I_{OFF} and (e) average I_{ON}/I_{OFF} ratio after being capped with 2.5/27.5 nm of AlO_x /HfO_x.

S.5.2 XPS Data after the Growth of 2.5/2.5 nm Bilayer Stacks on MoS_2

Figure S7 shows the S 2p, C 1s and Si 2p core level spectra after the growth of 2.5/2.5 nm AlO_x /HfO_x on MoS_2. The data are compared with respect to the 5 nm of HfO_x grown of MoS_2. The Si 2p spectrum is provided without any extra fitting because only the peak intensity was of interest.

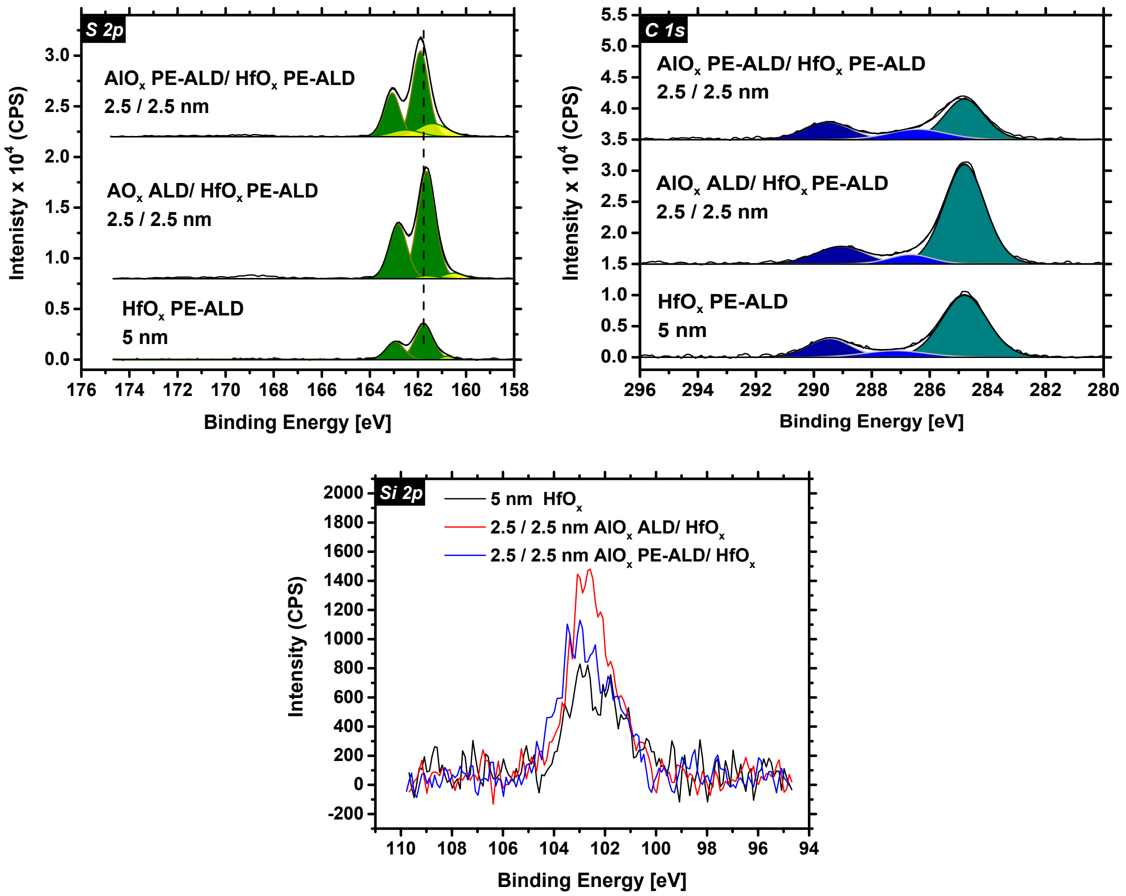

Figure S7. The S 2p, C 1s and Si 2p core level spectra, after the growth of 2.5/2.5 nm of AlO_x /HfO_x bilayer on MoS_2, with respect to 5 nm of HfO_x on MoS_2.

S.5.3 XPS Quantitative Details for 2.5/2.5 nm Bilayer Stacks

Table S3 provides the acquired data from the XPS analysis (Mo^{6+}/Mo^{4+}, Mo $3d_{5/2}$ and S $2p_{3/2}$), after the growth of 2.5/2.5 nm of AlO_x /HfO_x on MoS_2 films.

Table S3 CPS area under the Mo^{4+} and Mo^{6+} doublet peaks, their ratio as well as the Mo $3d_{5/2}$ and S $2p_{3/2}$ peak position and their binding energy shifts after the growth of 2.5/2.5 nm of AlO_x /HfO_x bilayers on MoS_2.

**MoS_2 Capping**	**Area CPS Mo^{4+}**	**Area CPS Mo^{6+}**	**$\frac{Mo^{6+}}{Mo^{4+}}$**	**Mo $3d_{5/2}$ peak [eV]**	**Mo 3d BE shifts [eV]**	**S 2p $_{3/2}$ peak [eV]**	**S 2p BE shifts [eV]**
**None**	132727	30031	0.22	229.8	0	162.6	0
**5 nm PE-ALD HfO_x**	12786	6710	0.52	228.9	-0.9	161.7	-0.9
**2.5/2.5 nm PE-ALD AlO_x /PE-ALD HfO_x**	31889	9931	0.31	229.0	-0.8	161.8	-0.8
**2.5/2.5 nm ALD AlO_x / PE-ALD HfOx**	39594	13047	0.32	228.8	-1.0	161.6	-1.0

S.5.4 HR-STEM Analysis

Figure S8 displays the HAADF-STEM images of 2.5/2.5 nm PE-ALD HfO_x/ALD AlO_x on MoS_2 in various magnification modes, to better visualize that the nucleation of the dielectric stack occurs initially at the grain boundaries and defect sites. The images also illustrate that 2.5/2.5 nm dielectric does not lead to a completely closed dielectric layer on the MoS_2 surface.

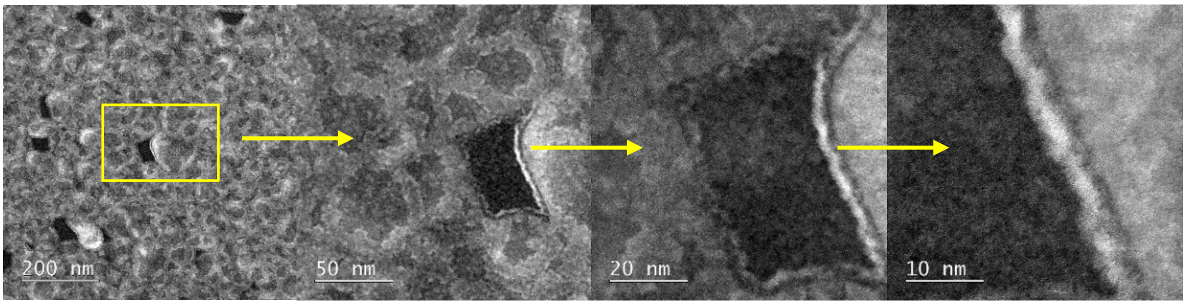

Figure S8. HAADF-STEM image of 2.5/2.5 nm PE-ALD HfO_x/ALD AlO_x on MoS_2 in various magnification modes for further visualizing that the dielectric nucleation initially starts at the grain boundaries and/or defects sites.

S.5.5 Average I_{ON} after the Growth of 5/25 nm AlO_x /HfO_x on MoS_2 FETs

As can be seen from **Figure S9**, it is only upon the growth of 5/25 nm ALD AlO_x/PE-ALD HfO_x on MoS_2 FETs that I_{ON} substantially improves and increases close to 1 µA/µm (on average and compared with the reference).

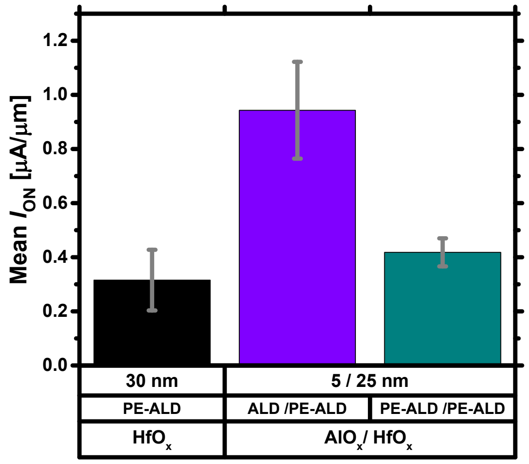

Figure S9. The average I_{ON} of the MoS_2 FETs capped with 5/25 nm AlO_x /HfO_x

S.5.6 Si 2p Spectrum of 5/2.5 nm AlO_x /HfO_x on MoS_2

One way to evaluate the growth rate of dielectrics on MoS_2 is to inspect the substrate Si 2p peak intensity. As can be seen in **Figure S10**, after the growth of 5/2.5 nm ALD AlO_x/PE-ALD HfO_x, the Si 2p peak intensity is still detectable, being as an indication that less bilayer dielectric is grown on MoS_2 when the AlO_x interlayer is processed by ALD.

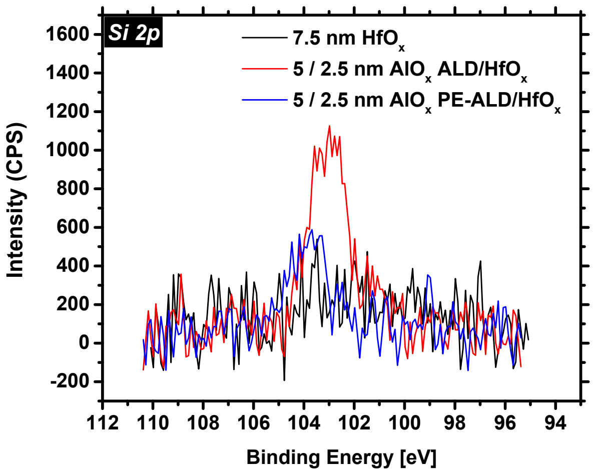

Figure S10. The Si 2p core level spectrum after the growth of 7.5 nm HfO_x and 5/2.5 nm of HfO_x/AlO_x bilayers on MoS_2.

S.5.7 XPS Quantitative Details for 5/2.5 nm Bilayer Cases

Table S4 provides the data acquired from XPS after the growth of 5/2.5 nm of AlO_x/HfO_x on MoS_2. The data reveals that 5 nm AlO_x interlayer grown by ALD or PE-ALD protects the MoS_2 surface from being oxidized upon the PE-ALD of HfO_x.

Table S4 The integrated area under the Mo^{4+} and Mo^{6+} doublet peaks and their ratio after the growth of 7.5 nm HfO_x and 5/2.5 nm AlO_x /HfO_x.

MoS_2 Capping	Thickness [nm]	Area CPS Mo^{4+}	Area CPS Mo^{6+}	Mo^{6+}/Mo^{4+}
**7.5 nm HfO_x**	7.5	8145	6125	0.75
**5/2.5 nm AlO_x ALD/HfO_x**	5/2.5	31645	7000	0.22
**5/2.5 nm AlO_x PE-ALD/HfO_x**	5/2.5	12018	2660	0.22

S.5.8 Device Metrics for Different AlO_x Interlayer Thicknesses

Figure S11 show the average I_{ON}, I_{OFF} and I_{ON}/I_{OFF} ratio of the MoS_2 FETs (capped with the AlO_x/HfO_x bilayers) and their dependence on the ALD AlO_x interlayer thickness.

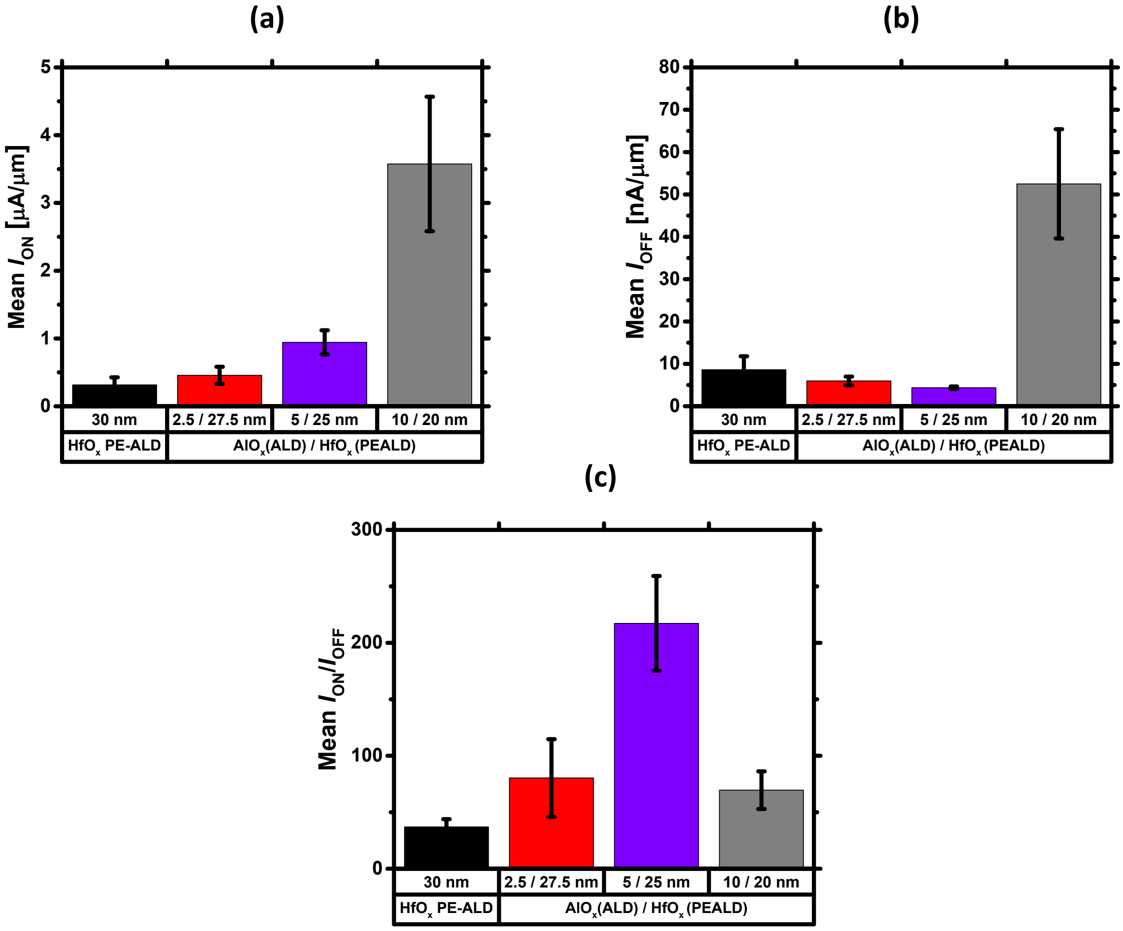

Figure S11. The average (a) I_{ON} (b) I_{OFF} (c) I_{ON}/I_{OFF} ratio for the MoS_2 FETs capped with bilayers of ALD AlO_x /PE-ALD HfO_x and their dependence on the ALD AlO_x interlayer thickness.

S.5.9 STEM Images of Different ALD AlO_x Interlayer Thicknesses Grown on MoS_2

Figure S12 displays the high angle annular dark field (HAADF)-STEM images of bilayer dielectrics grown on MoS_2 with various AlO_x interlayer thicknesses. As can be seen, by increasing the AlO_x interlayer thickness to 10 nm, a complete MoS_2 coverage can be eventually achieved.

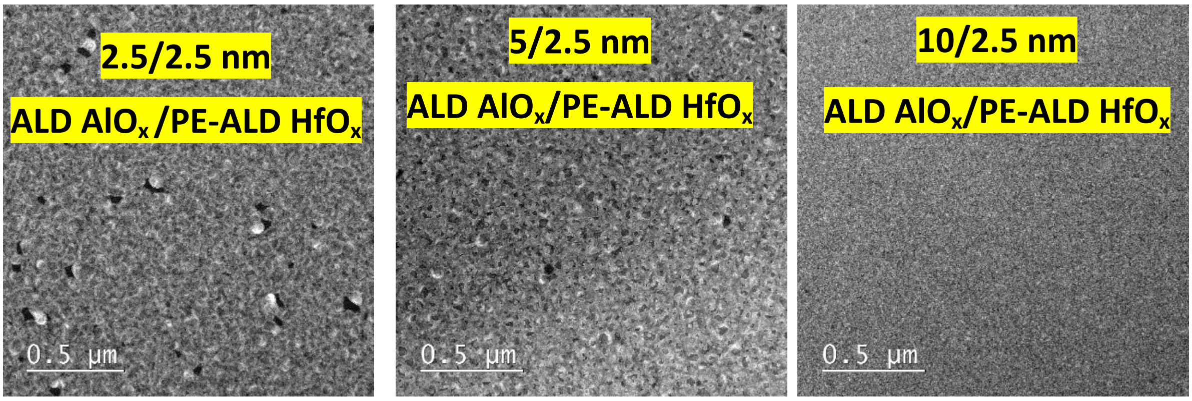

Figure S12. The HAADF STEM images of the ALD AlO_x /PEALD HfO_x on MoS_2 with various AlO_x interlayer thicknesses.

References

(1) Sharma, A.; Longo, V.; Verheijen, M. A.; Bol, A. A.; Kessels, W. M. M. (Erwin). Atomic Layer Deposition of HfO_2 Using $HfCp(NMe_2)_3$ and O_2 Plasma. *J. Vac. Sci. Technol. A Vacuum, Surfaces, Film.* **2017**, *35* (1), 01B130. https://doi.org/10.1116/1.4972210.

(2) Leonhardt, A.; Chiappe, D.; Afanas'ev, V. V.; El Kazzi, S.; Shlyakhov, I.; Conard, T.; Franquet, A.; Huyghebaert, C.; de Gendt, S. Material-Selective Doping of 2D TMDC through Al_xO_y Encapsulation. *ACS Appl. Mater. Interfaces* **2019**, *11* (45), 42697–42707. https://doi.org/10.1021/acsami.9b11550.
